# Use of an Automated Bilingual Digital Health Tool to Reduce Unhealthy Alcohol Use Among Latino Emergency Department Patients: A Randomized Clinical Trial

**DOI:** 10.1001/jamanetworkopen.2023.14848

**Published:** 2023-05-23

**Authors:** Federico E. Vaca, James Dziura, Fuad Abujarad, Michael Pantalon, Allen Hsiao, Jesse Reynolds, Kaitlin R. Maciejewski, Craig A. Field, Gail D’Onofrio

**Affiliations:** 1 Department of Emergency Medicine, University of California Irvine School of Medicine, Orange; 2 Department of Emergency Medicine, Yale School of Medicine, New Haven, Connecticut; 3 Department of Pediatrics, Section of Emergency Medicine, Yale School of Medicine, New Haven, Connecticut; 4 Department of Biostatistics, Yale School of Public Health, New Haven, Connecticut; 5 Latino Health Disparities Research, University of Texas at El Paso, El Paso

## Abstract

**Importance:**

Alcohol use disorders have a high disease burden among US Latino groups. In this population, health disparities persist, and high-risk drinking has been increasing. Effective bilingual and culturally adapted brief interventions are needed to identify and reduce disease burden.

**Objective:**

To compare the effectiveness of an automated bilingual computerized alcohol screening and intervention (AB-CASI) digital health tool with standard care for the reduction of alcohol consumption among US adult Latino emergency department (ED) patients with unhealthy drinking.

**Design, Setting, and Participants:**

This bilingual unblinded parallel-group randomized clinical trial evaluated the effectiveness of AB-CASI vs standard care among 840 self-identified adult Latino ED patients with unhealthy drinking (representing the full spectrum of unhealthy drinking). The study was conducted from October 29, 2014, to May 1, 2020, at the ED of a large urban community tertiary care center in the northeastern US that was verified as a level II trauma center by the American College of Surgeons. Data were analyzed from May 14, 2020, to November 24, 2020.

**Intervention:**

Patients randomized to the intervention group received AB-CASI, which included alcohol screening and a structured interactive brief negotiated interview in their preferred language (English or Spanish) while in the ED. Patients randomized to the standard care group received standard emergency medical care, including an informational sheet with recommended primary care follow-up.

**Main Outcomes and Measures:**

The primary outcome was the self-reported number of binge drinking episodes within the last 28 days, assessed by the timeline followback method at 12 months after randomization.

**Results:**

Among 840 self-identified adult Latino ED patients (mean [SD] age, 36.2 [11.2] years; 433 [51.5%] male; and 697 [83.0%] of Puerto Rican descent), 418 were randomized to the AB-CASI group and 422 to the standard care group. A total of 443 patients (52.7%) chose Spanish as their preferred language at enrollment. At 12 months, the number of binge drinking episodes within the last 28 days was significantly lower in those receiving AB-CASI (3.2; 95% CI, 2.7-3.8) vs standard care (4.0; 95% CI, 3.4-4.7; relative difference [RD], 0.79; 95% CI, 0.64-0.99). Alcohol-related adverse health behaviors and consequences were similar between groups. The effect of AB-CASI was modified by age; at 12 months, the relative reduction in the number of binge drinking episodes within the last 28 days in the AB-CASI vs standard care group was 30% in participants older than 25 years (RD, 0.70; 95% CI, 0.54-0.89) compared with an increase of 40% in participants 25 years or younger (RD, 1.40; 95% CI, 0.85-2.31; *P* = .01 for interaction).

**Conclusions and Relevance:**

In this study, US adult Latino ED patients who received AB-CASI had a significant reduction in the number of binge drinking episodes within the last 28 days at 12 months after randomization. These findings suggest that AB-CASI is a viable brief intervention that overcomes known procedural barriers to ED screening, brief intervention, and referral to treatment and directly addresses alcohol-related health disparities.

**Trial Registration:**

ClinicalTrials.gov Identifier: NCT02247388

## Key Points

**Question:**

Is a brief automated bilingual computerized alcohol screening and intervention (AB-CASI) health information technology tool superior to standard care in reducing alcohol consumption and alcohol-related adverse health behaviors and consequences among US Latino emergency department (ED) patients?

**Findings:**

In this randomized clinical trial of 840 self-identified adult Latino ED patients with unhealthy drinking, the number of binge drinking episodes within the last 28 days was significantly lower in the AB-CASI group compared with the standard care group (3.2 episodes vs 4.0; 7.7 episodes at baseline in both groups) at 12 months after randomization.

**Meaning:**

These findings suggest that AB-CASI is a viable approach to addressing alcohol-related health disparities in the ED, a setting that serves as a national health care front line and safety net.

## Teaser

This randomized clinical trial compares the effectiveness of an automated bilingual computerized alcohol screening and intervention with standard care for the reduction of alcohol use among US adult Latino emergency department patients with unhealthy drinking.

## Introduction

Alcohol use disorders (AUDs) are major contributors to US disease burden (eg, liver and cardiovascular diseases and cancers).^1,2^ In the US health care system, there are few settings in which patients with AUDs can be encountered more often than the emergency department (ED). Health disparities related to alcohol are also frequently observed in the ED. Data from the National Hospital Ambulatory Medical Care Survey^3^ reveal that in 2019, US EDs had more than 150 million visits. This number included more than 26 million ED visits by Latino patients; in the *chronic condition at ED visit* category, more than 5.1 million visits were categorized as *alcohol misuse, abuse, or dependence ED visits*.^3^ From 2006 to 2014, alcohol-related visits to EDs increased by 61.6%, including increases of more than 51% for acute alcohol-related visits and more than 75% for chronic alcohol-related visits.^4^ Furthermore, from 1999 to 2017, among those aged 16 years or older, national annual alcohol-related deaths doubled (from 35 914 to 72 558), and from 2015 to 2019, 20% of all alcohol-attributable deaths were among individuals aged 20 to 49 years.^5,6^

Conceptualization and testing of ED screening, brief intervention, and referral to treatment (ED-SBIRT) for alcohol use, which began more than 20 years ago, has provided an opportunity to identify and address patients with AUDs in the ED.^7^ Over time, ED-SBIRT has been evaluated and found to be associated with reductions in alcohol consumption as well as decreases in adverse physical and social consequences.^8,9,10,11^

High-quality systematic reviews^8,12,13^ have provided evidence to support screening and behavioral counseling interventions for unhealthy alcohol use, and the US Preventive Services Task Force has included this approach in its recommendations.^14^ A recent systematic review and meta-analysis^13^ reported that ED-SBIRT was associated with a small reduction in drinking, and its authors along with other experts^15,16^ made specific note of the potential population benefits of cumulative small individual reductions in alcohol use as a result of SBIRT efforts.

In the ED, routine implementation of SBIRT lags behind national guidelines.^17,18,19^ Perceived barriers to its regular implementation have included practitioner time burden, personnel cost, and maintenance of intervention fidelity. Moreover, the inability to deliver the intervention in the patient’s preferred language (eg, Spanish) is a fundamental rate-limiting step. Without language concordance, meaningful patient communication about unhealthy alcohol use and disease prevention is a failed endeavor. The inability to provide ED-SBIRT easily and readily in a language other than English limits the reach of prevention efforts. Furthermore, for US Latino patients, who represent the largest ethnic minority group (60.6 million individuals or 18.5% of the US population), this language barrier limits the ability of ED-SBIRT to address alcohol-related health disparities.^20^

Despite studies reporting that US Latinos are less likely to receive alcohol intervention,^21,22^ with few exceptions, most US ED-SBIRT clinical trials have excluded enrollment of predominantly Spanish-speaking patients. Research has revealed that daily heavy drinking and high rates of chronic liver disease and cirrhosis-related death are prevalent among Latino men who drink.^23,24,25,26,27,28,29,30,31^ While non-Latino White individuals are more likely to become dependent, once alcohol dependent, Latino individuals have a higher prevalence of recurrent or persistent dependence.^32,33,34^ Latino individuals also have higher rates of alcohol-related adverse consequences (eg, impaired driving).^35,36,37,38^ Because high-risk drinking among US Latino individuals has increased^39^ and related health disparities have persisted, and given the continued growth of the US Latino population (eg, from 2010 to 2019, 52% of 18.9 million residents added to the US population were of Latino ethnicity), a national research priority plan has been developed and disseminated.^20,40^ This plan was formulated from the existing literature to systematically address and more rapidly advance research in alcohol-related health disparities. The design and outcomes of the present randomized clinical trial (RCT) are highly relevant to this important research priority plan because they address screening and brief intervention methods for ethnic minority individuals and evaluate the effectiveness of a new tailored intervention among a large ethnic minority group and some of its subgroups. The objective of this RCT was to compare the effectiveness of our automated bilingual computerized alcohol screening and intervention (AB-CASI) digital health tool with standard care in reducing alcohol use among US adult Latino ED patients with unhealthy drinking.

## Methods

### Study Oversight and Ethics

This study was approved by the Human Investigation Committee of Yale School of Medicine (Supplement 1). All participants provided written informed consent in the language of their choice (English or Spanish). The study followed the Consolidated Standards of Reporting Trials (CONSORT) reporting guideline for RCTs.

### Design

We conducted a bilingual unblinded parallel-group RCT evaluating the effectiveness of AB-CASI in reducing alcohol use when compared with standard care among adult Latino ED patients. The trial was conducted from October 29, 2014, to May 1, 2020, and ended when recruitment goals were met. Data were analyzed from May 14, 2020, to November 24, 2020. During development of this trial, the *Diagnostic and Statistical Manual of Mental Disorders* (Fourth Edition; *DSM-IV*) was widely in use.^41^ The trial included the whole spectrum of unhealthy drinking, including individuals with drinking levels higher than the low-risk limits (ie, >3 drinks per occasion or >7 drinks per week for women and individuals aged >65 years; >4 drinks per occasion or >14 drinks per week for men) through individuals with alcohol dependence.^41^ The design of this RCT has been previously published in full detail.^42^

### Participants

Enrolled study participants were self-identified adult Latino ED patients (English or Spanish speaking) found to have unhealthy drinking. Exclusion criteria included current enrollment in a treatment program, pregnancy at enrollment, and the presence of conditions precluding the use of interviews or the AB-CASI tool (eg, suicidal or homicidal ideation or acute psychosis). The trial setting was the ED of a large urban community tertiary care center in the northeastern US that was verified as a level II trauma center by the American College of Surgeons.

### Interventions

#### AB-CASI

Patients randomized to the intervention group received AB-CASI, which is a bilingual (English or Spanish) digital health tool developed for automated ED-SBIRT encompassing the Alcohol Use Disorders Identification Test (AUDIT) and brief negotiation interview (BNI) with 4 core components: (1) raise subject, (2) provide feedback, (3) enhance motivation (with cultural considerations, such as emphasis of familismo [family]), and (4) negotiate advice.^43,44^ Using computer tablets (iPad 4th Generation; Apple Inc), AB-CASI was administered at the patient’s bedside by a trained bilingual research assistant. Participants selected their AB-CASI interface language, and questions and messages were displayed and spoken through headphones for privacy. The AB-CASI tool automatically collected demographic characteristics, administered the AUDIT, delivered a BNI (identifying quantity and frequency of alcohol use, readiness to change, reasons for reducing alcohol use, and goals) (eFigure in Supplement 2), and concluded with a printed personalized alcohol use reduction plan and counseling referral information. No modification to the AB-CASI tool was made throughout the course of the trial.

#### Standard Care

Participants not randomized to the AB-CASI group received standard emergency medical care, including an informational sheet with recommended primary care follow-up. Screening and referral requirements were performed according to American College of Surgeons level II trauma center designation guidelines. Social worker consultation was at the full discretion of the treating clinician.

### Procedures

Prospective participants were approached in the ED by trained bilingual and bicultural research assistants. Patients who self-identified as having Latino ethnicity received a health quiz (including questions about alcohol, tobacco, and seat belt use). Three standard questions about the quantity and frequency of alcohol use were asked: (1) on average, how many days per week do you drink alcohol? (2) on a typical day when you drink, how many drinks do you have? and (3) how many times in the past month have you had X [4 for women or 5 for men] or more drinks on any occasion? Patients reporting a consistently unhealthy pattern of drinking were approached for written informed consent in their language choice of English or Spanish.

### Baseline and Long-term Follow-up Assessments

Baseline and follow-up assessments (at 1 month, 6 months, and 12 months) included the AUDIT^45^ to measure alcohol use severity (administered at baseline); the timeline followback (TLFB) method to measure the number of binge drinking episodes and the number of weekly standard drinks within the last 28 days^46,47,48^ (administered at baseline, 1 month, 6 months, and 12 months); the Revised Injury Behavior Checklist^49^ to measure any physical injuries (administered at baseline and 12 months); the Short Inventory of Problems^50,51^ (administered at baseline, 1 month, 6 months, and 12 months) and brief event data^52,53^ report to measure alcohol-related adverse behaviors and consequences, such as driving after binge drinking, missing days of work, and getting arrested for any reason (administered at baseline, 1 month, 6 months, and 12 months); and the Treatment Services Review^54^ to measure receipt of specialized treatment services (administered at baseline, 1 month, 6 months, and 12 months). Bilingual supervised trained research assistants, blinded to group assignment, completed scripted follow-up assessments by telephone. Follow-up assessments lasted 15 to 20 minutes. Participants received gift cards ($20 at baseline, $25 at 1 month, $40 at 6 months, and $50 at 12 months).

### Outcomes

The primary outcome measure was the self-reported number of binge drinking episodes (defined as >3 standard drinks per occasion for women and individuals aged >65 years and >4 standard drinks per occasion for men) over the previous 28 days, assessed using 28-day TLFB at 12 months after randomization. We hypothesized that at 12 months, AB-CASI would be superior to standard care in reducing the number of binge drinking episodes and the mean number of weekly standard drinks over the last 28 days. Secondary outcomes included the self-reported mean number of weekly standard drinks measured by 28-day TLFB at 12 months after randomization and alcohol-related adverse health behaviors and consequences over the 12-month period. We hypothesized that at 12 months, AB-CASI would be superior to standard care in reducing alcohol-related adverse health behaviors and consequences.

### Sample Size

Sample size estimation was based on randomizing and following up a sufficient number of patients with unhealthy drinking to evaluate the primary hypothesis that AB-CASI would result in greater 12-month reductions in the primary outcome compared with standard care. An RCT by Fleming et al^55^ demonstrated that the number of binge drinking episodes in the past 30 days was reduced by 1.14 in the intervention compared with the control condition. D’Onofrio et al^56^ reported similar findings in an RCT conducted among individuals with hazardous and harmful drinking. Given a power of 80%, a significance level of 2-sided α = .05, an SD of 5.2 for the number of binge drinking episodes in the past 28 days, and 1:1 intervention allocation, a sample of 327 participants per group was required to detect a 1.14-episode difference between AB-CASI and standard care. A total of 840 patients with unhealthy drinking were enrolled and randomized to accommodate a study withdrawal rate of up to 20%.

### Randomization

Participants were randomized 1:1 using a stratified randomization procedure implemented by the OnCore Clinical Trials Management System (Forte Research). Stratification factors were alcohol use severity measured by AUDIT score (range, 0-40, with <20 indicating alcohol nondependence and ≥20 indicating alcohol dependence)^45^ and preferred language. The statistician (Dr Dziura) used a computer algorithm to generate a permuted block randomization sequence. The sequence was concealed in the OnCore system and allocated after enrollment of a participant by a trained bilingual research assistant.

### Statistical Analysis

Analyses were conducted according to the intention-to-treat principle and performed using SAS software, version 9.4 (SAS Institute Inc) (Supplement 1). A repeated-measures generalized linear mixed model (GLMM) with negative binomial distribution was used to estimate primary outcome differences between the AB-CASI and standard care groups. The mixed model jointly modeled the number of binge drinking episodes at baseline, 1 month, 6 months, and 12 months. This model fit the same mean to both treatment groups at baseline, thereby conditioning (ie, adjusting) the estimates of treatment effects on the baseline number of binge drinking episodes.^57^ The model included fixed effects for intervention, time, and the interaction of intervention with time. Additional fixed effects were included for baseline covariates (sex, preferred language, and alcohol dependence status). Analysis permitted the inclusion of all randomized participants and assumed that missing data occurred at random. Inclusion of baseline, 1-month, 6-month, and 12-month outcome data in the model assisted in meeting this assumption. Linear contrasts with a significance level of 2-sided *P* = .05 were used to estimate intervention group differences and 95% CIs at 1 month, 6 months, and 12 months. Because the negative binomial model was multiplicative, the relative difference (RD) between groups (ie, the ratio of the mean number of binge drinking episodes within the last 28 days in the AB-CASI group to the mean number in the standard care group) with 95% CIs was reported.

Similar repeated-measures mixed-model analyses were implemented for each secondary outcome. Comparison of all secondary outcomes between groups was evaluated at a significance level of 2-sided *P* = .01 to control inflated type 1 errors from multiple significance testing.

Along with stratification factors, the heterogeneity of treatment effects on the primary outcome was assessed for subgroups based on prespecified factors assessed at baseline (eg, age, sex, biculturalism score, and primary reason for ED visit). The biculturalism score (which includes Hispanicism and Americanism scores) was measured by the Bicultural Involvement Questionnaire–Short Version^58^; biculturalism, Hispanicism, and Americanism are categories approximating acculturation referring to respective levels of comfort and involvement in activities that are classified as *more American* or *more Hispanic or Latino*. These exploratory subgroup analyses were conducted within the GLMM framework by including the interactions of treatment, time, and subgroup factors. Differences between AB-CASI and standard care were calculated for each level of the subgroup and were compared using linear contrasts.

## Results

### Study Population

**Figure 1.**
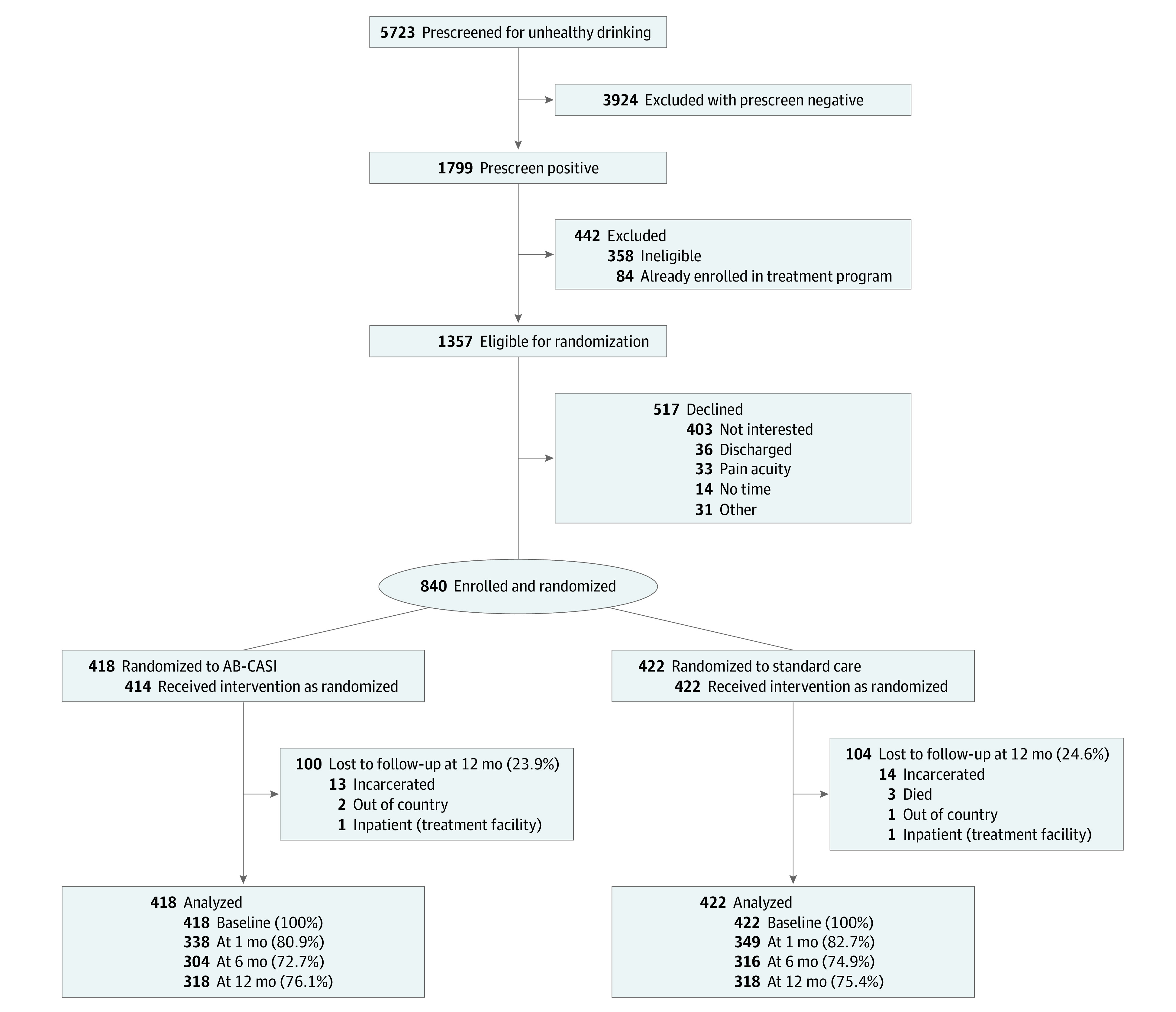
Study Flowchart AB-CASI indicates automated bilingual computerized alcohol screening and intervention.

Of 1799 Latino ED patients who screened positive for unhealthy drinking, 1357 met eligibility criteria. A total of 840 patients (61.9%) were enrolled and randomized; of those, 418 patients received AB-CASI and 422 received standard care (Figure 1). Four participants randomized to the AB-CASI group did not receive the intervention. Among 840 randomized participants, the mean (SD) age was 36.2 (11.2) years; 433 (51.5%) were male, 407 (48.5%) were female, 152 (18.1%) were assessed as having alcohol dependence, and 443 (52.7%) chose Spanish as their preferred language. Most participants self-identified as Puerto Rican (697 [83.0%]), were born on the US mainland (456 [54.3%]), and had health insurance (556 [66.2%]). The mean (SD) time to complete AB-CASI was 8 (5) minutes in the English language and 10 (6) minutes in the Spanish language. Baseline characteristics were similar between the AB-CASI and standard care groups (Table 1). Most patients in the AB-CASI group (220 [52.6%] chose to receive the intervention in the Spanish language.

**Table 1.** Participant Baseline Characteristics

Characteristic	Participants, No. (%)	
	AB-CASI group (n = 418)	Standard care group (n = 422)
Age, mean (SD), y	37.0 (11.5)	35.5 (11.0)
Sex		
Female	206 (49.3)	201 (47.6)
Male	212 (50.7)	221 (52.4)
AUDIT score, mean (SD)^a^	12.1 (7.8)	11.9 (8.0)
Alcohol dependence	76 (18.2)	76 (18.0)
Preferred language		
English	198 (47.4)	199 (47.2)
Spanish	220 (52.6)	223 (52.8)
Race		
American Indian or Alaska native	2 (0.5)	1 (0.2)
Black or African American	14 (3.3)	26 (6.2)
Native Hawaiian or other Pacific Islander	2 (0.5)	0
White	50 (12.0)	68 (16.1)
Other	347 (83.0)	321 (76.1)
No answer provided	3 (0.7)	6 (1.4)
Hispanic origin^b^		
Central American	8 (1.9)	14 (3.3)
Cuban	0	1 (0.2)
Dominican	18 (4.3)	28 (6.6)
Mexican	24 (5.7)	19 (4.5)
Puerto Rican	352 (84.2)	345 (81.8)
South American	8 (1.9)	8 (1.9)
Other	2 (0.5)	3 (0.7)
No answer provided	6 (1.4)	4 (0.9)
Nativity		
US mainland	218 (52.2)	238 (56.4)
Other US territory or country	196 (46.9)	175 (41.5)
No answer provided	4 (1.0)	9 (2.1)
Marital status		
Divorced or separated	81 (19.4)	76 (18.0)
Married	71 (17.0)	54 (12.8)
Single or never married	226 (54.1)	255 (60.4)
Widowed	7 (1.7)	1 (0.2)
Other	27 (6.5)	30 (7.1)
No answer provided	6 (1.4)	6 (1.4)
Biculturalism score, mean (SD)^c^	13.2 (29.0)	9.9 (28.5)
Hispanicism score, mean (SD)^c^	61.6 (13.5)	61.0 (14.2)
Americanism score, mean (SD)^c^	48.4 (21.0)	51.1 (20.4)
Total cultural involvement score, mean (SD)	109.9 (20.2)	112.1 (20.5)
Educational level		
Less than high school	173 (41.4)	163 (38.6)
High school or some college	205 (49.0)	214 (50.7)
Associate’s degree or higher	31 (7.4)	38 (9.0)
Other	7 (1.7)	5 (1.2)
No answer provided	2 (0.5)	2 (0.5)
Has a usual physician	220 (52.6)	225 (53.3)
Health insurance^b^		
Disability or government services	7 (1.7)	9 (2.1)
Medicaid	293 (70.1)	313 (74.2)
Medicare	23 (5.5)	22 (5.2)
Private PPO	27 (6.5)	16 (3.8)
Other	1 (0.2)	0
None	142 (34.0)	142 (33.6)
Current smoking	235 (56.2)	237 (56.2)

Abbreviations: AB-CASI, automated bilingual computerized alcohol screening and intervention; AUDIT, Alcohol Use Disorders Identification Test; PPO, preferred provider organization.

^a^ Score range, 0-40, with <20 indicating alcohol nondependence and ≥20 indicating alcohol dependence.

^b^ Multiple categories could be selected.

^c^ Measured by the Bicultural Involvement Questionnaire–Short Version.^58^ Biculturalism, Hispanicism, and Americanism are categories approximating acculturation referring to respective levels of comfort and involvement in activities that are classified as *more American* or *more Hispanic or Latino*.

### Primary Outcome

In the AB-CASI group, the GLMM analysis revealed that the mean number of binge drinking episodes within the last 28 days was 7.7 (95% CI, 6.9-8.7) at baseline and decreased to 3.5 (95% CI, 3.0-4.2) at 1 month, 3.4 (95% CI, 2.9-4.1) at 6 months, and 3.2 (95% CI, 2.7-3.8) at 12 months (Table 2). In the standard care group, the mean number of binge drinking episodes was 7.7 (95% CI, 6.9-8.7) at baseline, decreasing to 3.9 (95% CI, 3.3-4.6) at 1 month, 3.1 (95% CI, 2.6-3.7) at 6 months, and 4.0 (95% CI, 3.4-4.7) at 12 months. The number of binge drinking episodes within 28 days at 12 months after randomization (primary outcome) was significantly lower in those receiving AB-CASI vs standard care (RD, 0.79; 95% CI, 0.64-0.99). The RD of 0.79 indicates that the mean number of binge drinking episodes within the last 28 days in the AB-CASI group was 79% of the mean number in the standard care group. The number of binge drinking episodes within 28 days did not significantly differ between groups at 1 month and 6 months (Table 2).

**Table 2.** Primary and Secondary Outcomes: Estimated Means and Comparisons of AB-CASI vs Standard Care From the Generalized Linear Mixed Model^a^

Outcome	Mean (95% CI)		Ratio (95% or 99% CI)^b^	*P* value
	AB-CASI group	Standard care group		
**No. of binge drinking episodes in last 28 d** ^c^ **^,^** ^d^ **^,^** ^e^				
1 mo	3.5 (3.0-4.2)	3.9 (3.3-4.6)	0.90 (0.73-1.11)	.34
6 mo	3.4 (2.9-4.1)	3.1 (2.6-3.7)	1.09 (0.88-1.37)	.43
12 mo	3.2 (2.7-3.8)	4.0 (3.4-4.7)	0.79 (0.64-0.99)	.04
**No. of weekly standard drinks in last 28 d** ^c^ **^,^** ^d^				
1 mo	12.4 (10.8-14.1)	12.2 (10.7-13.9)	1.01 (0.81-1.25)	.90
6 mo	11.6 (10.1-13.4)	10.5 (9.1-12.1)	1.11 (0.88-1.40)	.25
12 mo	10.0 (8.6-11.5)	12.3 (10.7-14.1)	0.81 (0.64-1.02)	.02
**Driven a car, truck, motorcycle, or boat after binge drinking in last 30 d, over past 6 mo** ^c^ **^,^** ^e^ **^,^** ^f^				
1 mo	0.02 (0.01-0.05)	0.03 (0.01-0.05)	0.92 (0.26-3.18)	.85
6 mo	0.07 (0.04-0.12)	0.07 (0.04-0.12)	1.00 (0.42-2.40)	.99
12 mo	0.05 (0.03-0.09)	0.05 (0.03-0.09)	1.09 (0.41-2.89)	.83
**Missed full or partial day of work in last 30 d, over past 6 mo** ^f^ **^,^** ^g^				
1 mo	0.06 (0.04-0.11)	0.08 (0.05-0.13)	0.79 (0.33-1.87)	.48
6 mo	0.07 (0.04-0.12)	0.07 (0.04-0.12)	1.01 (0.41-2.48)	.99
12 mo	0.07 (0.04-0.12)	0.06 (0.03-0.10)	1.17 (0.47-2.95)	.66
**Arrested for anything in last 30 d, over past 6 mo** ^f^ **^,^** ^g^				
1 mo	0.004 (0-0.03)	0.02 (0.01-0.05)	0.19 (0.01-3.29)	.13
6 mo	0.03 (0.01-0.08)	0.03 (0.01-0.08)	1.02 (0.24-4.28)	.98
12 mo	0.04 (0.02-0.09)	0.06 (0.03-0.11)	0.73 (0.22-2.43)	.50
**Any reported injuries in last 6 mo** ^h^ **^,^** ^i^				
12 mo	0.13 (0.07-0.24)	0.16 (0.09-0.27)	0.85 (0.41-1.77)	.58
**Any reported injuries while driving in last 6 mo** ^h^ **^,^** ^i^				
12 mo	0.01 (0.001-0.10)	0.03 (0.004-0.20)	0.38 (0.04-3.43)	.26
**Any reported injuries requiring a physician in last 6 mo** ^h^ **^,^** ^i^				
12 mo	0.09 (0.04-0.19)	0.11 (0.05-0.22)	0.84 (0.33-2.16)	.63
**Any reported injuries when alcohol was consumed 2 h earlier in last 6 mo** ^h^ **^,^** ^i^				
12 mo	0.06 (0.03-0.16)	0.10 (0.04-0.22)	0.63 (0.18-2.18)	.34

Abbreviation: AB-CASI, automated bilingual computerized alcohol screening and intervention.

^a^ The primary outcome was the self-reported number of binge drinking episodes in the last 28 days, assessed at 12 months after randomization. All other outcomes were secondary.

^b^ For count outcomes (binge drinking episodes and weekly standard drinks), least squares means were used to calculate relative difference, which represents the ratio of the mean score of the AB-CASI group to the mean score of the standard care group (ie, mean score in the AB-CASI group divided by mean score in the standard care group). For binary outcomes (driven, missed work, arrested, and reported injuries), estimated probabilities were used to calculate risk ratio. 95% CIs were used for the number of binge drinking episodes, and 99% CIs were used for all other outcomes.

^c^ Adjusted for baseline number of binge drinking episodes, sex, preferred language (English or Spanish), and alcohol dependence status.

^d^ Based on the timeline followback method.^46,47,48^

^e^ Binge drinking was defined as 4 or more drinks per occasion for women or individuals older than 65 years and 5 or more drinks per occasion for men.

^f^ Based on the brief event data measure.^52,53^

^g^ Adjusted for baseline number of binge drinking episodes, baseline number of weekly standard drinks, sex, preferred language (English or Spanish), and alcohol dependence status. Missing data were recoded as a response of *no*.

^h^ Adjusted for baseline outcome, baseline number of binge drinking episodes, baseline number of weekly standard drinks, sex, preferred language (English or Spanish), and alcohol dependence status.

^i^ Based on the Revised Injury Behavior Checklist.^49^

### Secondary Outcomes

In the AB-CASI group, the mean number of weekly standard drinks decreased from 22.8 (95% CI, 20.8-25.1) at baseline to 12.4 (95% CI, 10.8-14.1) at 1 month, 11.6 (95% CI, 10.1-13.4) at 6 months, and 10.0 (95% CI, 8.6-11.5) at 12 months (Table 2). The standard care group reported a mean number of weekly standard drinks of 22.8 (95% CI, 20.8-25.1) at baseline, 12.2 (95% CI, 10.7-13.9) at 1 month, 10.5 (95% CI, 9.1-12.1) at 6 months, and 12.3 (95% CI, 10.7-14.1) at 12 months. At 12 months, the mean number of weekly standard drinks was 19% lower in the AB-CASI group compared with the standard care group (RD, 0.81; 99% CI, 0.64-1.02), but this difference was not statistically significant at the prespecified level of *P* = .01 for secondary outcomes. Alcohol-related adverse health behaviors and consequences over 12 months were similar between groups (Table 2).

### Exploratory Subgroup Analyses

**Figure 2.**
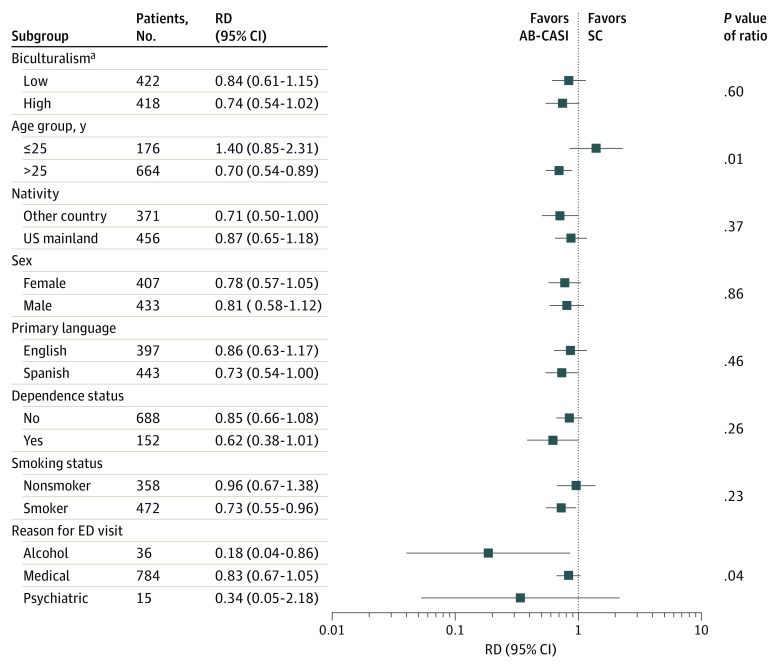
Effect Modifier Analysis of Number of Binge Drinking Episodes at 12 Months The relative difference (RD) represents the ratio of the mean number of binge drinking episodes in the last 28 days in the automated bilingual computerized alcohol screening and intervention (AB-CASI) group to the mean number in the standard care (SC) group. ED indicates emergency department. ^a^For the biculturalism score (which was measured by the Bicultural Involvement Questionnaire–Short Version^58^ and includes Hispanicism and Americanism scores), the low range is −80.00 to 5.00, and the high range is 5.74 to 80.00. Biculturalism, Hispanicism, and Americanism are categories approximating acculturation referring to respective levels of comfort and involvement in activities that are classified as *more American* or *more Hispanic or Latino*.

At 12 months, the effect of AB-CASI on the number of binge drinking episodes within the last 28 days was modified by age and primary reason for ED visit. In those older than 25 years, binge drinking episodes were 30% lower (RD, 0.70; 95% CI, 0.54-0.89) vs those 25 years or younger, for whom the point estimate for binge drinking episodes was 40% higher (RD, 1.40; 95% CI, 0.85-2.31; *P* = .01 for interaction) in the AB-CASI group vs the standard care group, although 95% CIs were wide due to the small size of the subgroup 25 years or younger (n = 176) (Figure 2). Furthermore, the magnitude of the AB-CASI reduction was greater in participants with an ED visit for a primary alcohol-related reason (RD, 0.18; 95% CI, 0.04-0.86) vs a primary medical-related reason (RD, 0.83; 95% CI, 0.67-1.05) or a primary psychiatric-related reason (RD, 0.34; 95% CI, 0.05-2.18; *P* = .04 for interaction). No intervention-related serious adverse outcomes were encountered.

## Discussion

To our knowledge, this study is the first large US RCT of ED-SBIRT using a bilingual automated digital health tool to show effectiveness in reducing unhealthy alcohol use. The AB-CASI tool required little time to administer (8-10 minutes, replicated in this trial)^59,60^ and effectively identified patients with unhealthy drinking while providing tailored bilingual ED-SBIRT. The AB-CASI tool outperformed standard care specifically for reduction in the mean number of binge drinking episodes within 28 days by 21%. Although the mean number of weekly standard drinks was 19% lower in the AB-CASI group at 12 months, the difference did not meet the prespecified threshold for significance. However, while both groups had reductions in alcohol use at 1 month, which could suggest an effect because of the need to initially seek emergency care in itself, the reduction observed in the AB-CASI group endured at 12 months. Identifying that the AB-CASI group had nearly 1 less binge drinking episode than the standard care group is clinically meaningful and worthy of advocating for routine ED screening. This reduction is of particular note when considering that 1 binge drinking episode entails consuming several drinks over a short time, and binge drinking has a well-known association with other health-risking behaviors and a higher risk of acute toxic exposure to end organs. More than one-half (52.6%) of the participants chose to receive AB-CASI in Spanish. In the current state of national ED-SBIRT implementation, it is plausible that this specific vulnerable group would not have received intervention due to the limitations of ED-SBIRT delivery in Spanish and intervention knowledge.

The AB-CASI tool can provide an effective and cost-feasible strategy to address alcohol-related health disparities.^20^ While studies have reported increases in alcohol use and misuse among US Latino individuals,^31,39^ they have also highlighted continued challenges in receiving intervention and treatment, which are magnified for monolingual US Latino immigrants.^22,61,62^ Given our findings, it is important to keep in mind the heterogeneity of the US Latino population. Moreover, it has been well established that drinking patterns in this population are also heterogeneous, having implications for the treatment and recovery of those with AUDs.^32,63,64^ Previous studies^63,64^ have found that Puerto Rican women who drink have the highest binge drinking rates among all US Latina women. We found no differential intervention effect among men and women, showing that AB-CASI was equally effective. This finding indicates relevant benefits at a population level when considering sex differences in AUDs, particularly because the alcohol use and misuse gap between men and women continues to narrow, with detriment to women.^65,66,67,68^ While exploratory, we observed a significantly greater reduction in the number of binge drinking episodes within the last 28 days among those in the AB-CASI group who were older than 25 years compared with those who were 25 years or younger. Given the smaller subgroup of participants 25 years or younger (n = 176), further investigation of the impact of the intervention in younger individuals with unhealthy drinking is warranted. We also observed greater reductions for those with a primary alcohol-related reason for visiting the ED. Taken together, these findings can inform ED-SBIRT programs and research addressing alcohol-related health disparities among Latino ED patients with unhealthy drinking.^20^

Previous ED-SBIRT studies^69,70^ bolster evidence for cost savings and improved health. A study by Barbosa et al^69^ evaluated the cost-effectiveness of delivering ED-SBIRT and compared it with SBIRT delivery in other outpatient medical settings. The ED was found to be the most cost-effective delivery site based on dollars paid per quality-adjusted life years gained (ie, improved health). Furthermore, ED-SBIRT provided superior effectiveness at lower cost, with larger reductions in social cost. A recent study^70^ found that ED-SBIRT can be an effective cost-reducing strategy to address unhealthy alcohol use, and the authors advocated for policy makers and payers to prioritize these types of interventions.

### Limitations

This study has limitations. First, trial enrollment occurred only at 1 ED in a large urban community tertiary care center in the northeastern US. Second, enrolled participants were predominantly of Puerto Rican descent. While not surprising given the geographic location of the enrollment site and the historic predominance of the Puerto Rican population compared with other Latino groups in the northeastern US, consideration of variability in cultural stressors within different US Latino subgroups could be relevant to interpretation of the findings. Third, while our participant retention was successful, we are not able to comprehensively account for all outcomes among those unavailable for follow-up at some point after the initial baseline assessment. Given the short engagement with the interventions, it is unlikely that mechanisms leading to study withdrawal and missing data months after the intervention were differential between the 2 groups. Rates and reasons for missing outcomes were similar across intervention groups at all time points. Fourth, this trial was originally designed before 2013 and conducted using long-standing *DSM-IV* criteria of AUDs. In mid-2013, the *Diagnostic and Statistical Manual of Mental Disorders* (Fifth Edition; *DSM-5*)^71^ was released with different AUD terms and threshold criteria. While extensive overlap exists between the *DSM-IV* and *DSM-5*, being informed of the criteria differences would be prudent when interpreting trial findings.

## Conclusions

In this RCT, the use of AB-CASI in the ED was effective in reducing alcohol use among US English- and Spanish-speaking Latino patients who were identified along the spectrum of unhealthy alcohol use. Highly acceptable to patients and brief in delivery, the AB-CASI tool overcame several long-reported barriers to ED-SBIRT, successfully addressing alcohol-related health disparities among the growing US Latino population.

## Notes

Supplement 1.
Trial Protocol and Statistical Analysis Plan

Supplement 2. **eFigure.** Example Screenshots of AB-CASI: English and Spanish Versions

Supplement 3.
Data Sharing Statement
