# Supplementary material for: Use of an Automated Bilingual Digital Health Tool to Reduce Unhealthy Alcohol Use Among Latino Emergency Department Patients: A Randomized Clinical Trial
Source: JAMA Netw Open. 2023 May 23;6(5):e2314848. doi: 10.1001/jamanetworkopen.2023.14848 (PMC10208138; doi:10.1001/jamanetworkopen.2023.14848)
Supplement: Supplement 1. — Trial Protocol and Statistical Analysis Plan [file jamanetwopen-e2314848-s001.pdf]

YALE UNIVERSITY
HUMAN INVESTIGATION COMMITTEE

Application to Involve Human Subjects in Biomedical Research
100 FR1 (2013-1)

Please refer to the HIC website for application instructions and information required to complete this application. The Instructions are available at http://www.yale.edu/hrpp/forms-templates/biomedical.html<br>Submit the original application and one (1) copy of all materials including relevant sections of the grant which funds this project (if applicable) to the HIC.	HIC OFFICE USE ONLY	

SECTION I: ADMINISTRATIVE INFORMATION

**Title of Research Project: Title of Research Project: Automated Bilingual Computerized Alcohol Screening and Intervention in Latinos (AB-CASI).**			
**Principal Investigator: Federico Vaca, M.D.**	**Yale Academic Appointment: Professor, Department of Emergency Medicine**		
**Department: Department of Emergency Medicine**			
**Campus Address: Department of Emergency Medicine<br>464 Congress Avenue-Suite 260.**			
**Campus Phone: 203-785-4363**	**Fax: 203-785-4580**	**Pager:**	**E-mail:federico.vaca@yale.edu**
**Protocol Correspondent Name & Address (*if different than PI*): Alexei Nelayev**			
**Campus Phone: 203-737-6152**	**Fax: 203-785-4580**	**E-mail: alexei.nelayev@yale.edu**	
**Yale Cancer Center CTO Protocol Correspondent Name & Address (*if applicable*):**			
**Campus Phone:**	**Fax:**	**E-mail:**	
**Business Manager:**			
**Campus Phone :**	**Fax :**	**E-mail**	

**Faculty Advisor:**(required if PI is a student, resident, fellow or other trainee) ☐ **NA**	**Yale Academic Appointment:**
**Campus Address:**	

Campus Phone:	Fax:	Pager:	E-mail:

Investigator Interests:

Does the principal investigator, or do any research personnel who are responsible for the design, conduct or reporting of this project or any of their family members (spouse or dependent child) have an incentive or interest, financial or otherwise, that may affect the protection of the human subjects involved in this project, the scientific objectivity of the research or its integrity? Note: The Principal Investigator (Project Director), upon consideration of the individual's role and degree of independence in carrying out the work, will determine who is responsible for the design, conduct, or reporting of the research.

See Disclosures and Management of Personal Interests in Human Research http://www.yale.edu/hrpp/policies/index.html#COI

 Yes • No

Do you or does anyone on the research team who is determined by you to be responsible for the design, conduct or reporting of this research have any patent (sole right to make, use or sell an invention) or copyright (exclusive rights to an original work) interests related to this research protocol?

 Yes •No

If yes to either question above, list names of the investigator or responsible person:

The Yale University Principal Investigator, all Yale University co-investigators, and all Yale University individuals who are responsible for the design, conduct or reporting of research must have a current financial disclosure form on file with the University's Conflict of Interest Office. Yale New Haven Hospital personnel who are listed as con-investigators on a protocol with a Yale University Principal Investigator must also have a current financial disclosure form on file with the University's Conflict of Interest Office. If this has not been done, the individual(s) should follow this link to the COI Office Website to complete the form: http://www.yale.edu/coi/

NOTE: The requirement for maintaining a current disclosure form on file with the University's Conflict of Interest Office extends primarily to Yale University and Yale-New Haven Hospital personnel. **Whether or not they are required to maintain a disclosure form with the University's Conflict of Interest Office, all investigators and individuals deemed otherwise responsible by the PI who are listed on the protocol are required to disclose to the PI any interests that are specific to this protocol.**

SECTION II: GENERAL INFORMATION

1. **Performing Organizations:** Identify the hospital, in-patient or outpatient facility, school or other agency that will serve as the location of the research. Choose all that apply:

a. Internal Location[s] of the Study:

☐ Magnetic Resonance Research Center (MR-TAC)
☐ Yale Cancer Center/Clinical Trials Office (CTO)
☐ Yale Cancer Center/Smilow
☐ Yale-New Haven Hospital
☐ Cancer Data Repository/Tumor Registry
☒ Specify Other Yale Location: Bridgeport Hospital ED in Bridgeport, Connecticut.
☐ Yale University PET Center
☐ YCCI/Church Street Research Unit (CSRU)
☐ YCCI/Hospital Research Unit (HRU)
☐ YCCI/Keck Laboratories
☐ Yale-New Haven Hospital—Saint Raphael Campus

b. External Location[s]:
☐ APT Foundation, Inc.
☐ Connecticut Mental Health Center
☐ Clinical Neuroscience Research Unit (CNRU)
☐ Other Locations, Specify:
☐ Haskins Laboratories
☐ John B. Pierce Laboratory, Inc.
☐ Veterans Affairs Hospital, West Haven
☐ International Research Site (Specify location(s)):

c. Additional Required Documents *(check all that apply)*: ☐ N/A
☐ *YCCI-Scientific and Safety Committee (YCCI-SSC) Approval Date:
☐ *Pediatric Protocol Review Committee (PPRC) Approval Date:
☐ *YCC Protocol Review Committee (YRC-PRC) Approval Date:
☐ *Dept. of Veterans Affairs, West Haven VA HSS Approval Date:
☐ *Radioactive Drug Research Committee (RDRC) Approval Date:
☐ YNHH-Radiation Safety Committee (YNHH-RSC) Approval Date:
☐ Magnetic Resonance Research Center PRC (MRRC-PRC) Approval Date:
☐ YSM/YNHH Cancer Data Repository (CaDR) Approval Date:
☐ Dept. of Lab Medicine request for services or specimens form
☐ Imaging on YNHH Diagnostic Radiology equipment request form (YDRCTO request) found at http://radiology.yale.edu/research/ClinTrials.aspx)
Approval from these committees is required before final HIC approval is granted. See instructions for documents required for initial submission and approval of the protocol. Allow sufficient time for these requests. Check with the oversight body for their time requirements.

2. **Probable Duration of Project:** State the expected duration of the project, including all follow-up and data analysis activities.

   Probable duration of the project is six years.

3. **Research Type/Phase: (Check all that apply)**
   a. **Study Type**
   ☒ Single Center Study
   ☐ Multi-Center Study
   Does the Yale PI serve as the PI of the multi-site study? Yes ☐ No ☐
   ☐ Coordinating Center/Data Management
   ☐ Other:

   b. **Study Phase** ☒ **N/A**

APPROVED BY THE YALE UNIVERSITY IRB 3/23/2020

☐ Pilot ☐ Phase I ☐ Phase II ☐ Phase III ☐ Phase IV
☐ Other (*Specify*)

4. **Area of Research: (Check all that apply)** Note that these are overlapping definitions and more than one category may apply to your research protocol. Definitions for the following can be found in the instructions section 4c:

☒ Clinical Research: Patient-Oriented
☐ Clinical Research: Epidemiologic and Behavioral
☐ Translational Research #1 ("Bench-to-Bedside")
☐ Translational Research #2 ("Bedside-to-Community")
☐ Clinical Research: Outcomes and Health Services
☐ Interdisciplinary Research
☐ Community-Based Research

5. Is this study a clinical trial? Yes ☒ No ☐

NOTE the current ICMJE (International Committee of Medical Journal Editors) definition of a clinical trial: "any research study that prospectively assigns human participants or groups of humans to one or more health-related interventions to evaluate the effects on health outcomes." Health-related interventions include any intervention used to modify a biomedical or health-related outcome (for example, drugs, surgical procedures, devices, behavioral treatments, dietary interventions, and process-of-care changes). Health outcomes include any biomedical or health-related measures obtained in patients or participants, including pharmacokinetic measures and adverse events"

If yes, where is it registered? The study has not been registered with the clinicaltrials.gov registry. However, the PI will register the study prior to enrolling subjects.

Clinical Trials.gov registry ☐
Other (*Specify*)

Registration of clinical trials ***at their initiation*** *is required by the FDA, NIH and by the ICMJE.*

If this study is registered on clinicaltrials.gov, there is new language in the consent form and compound authorization that should be used.

For more information on registering clinical trials, including whether your trial must be registered, see the YCCI webpage, http://ycci.yale.edu/researchers/ors/registerstudy.aspx or contact YCCI at 203.785.3482)

6. Does the Clinical Trials Agreement (CTA) require compliance with ICH GCP (E6)?
Yes ☐ No☒

7. Will this study have a billable service? A Billable Service is defined as a service or procedure that will be ordered, performed or result in charging in EPIC for individuals who are enrolled in a clinical research study, regardless if the charge is intended to be paid by the subject/their insurance or the research study.

Yes ☐ No☒

If you answered "yes", this study will need to be set up in OnCore Support

http://medicine.yale.edu/ymg/systems/ppm/index.aspx

8.. Are there any procedures involved in this protocol that will be performed at YNHH or one of its affiliated entities? Yes ___ No __X_ *If Yes, please answer questions a through c and note instructions below. If No, proceed to Section III.*
a. Does your YNHH privilege delineation currently include the **specific procedure** that you will perform?
b. Will you be using any new equipment or equipment that you have not used in the past for this procedure?

c. Will a novel approach using existing equipment be applied?

If you answered "no" to question 7a, or "yes" to question 7b or c, please contact the YNHH Department of Physician Services (688-2615) for prior approval before commencing with your research protocol.

SECTION III: FUNDING, RESEARCH TEAM AND TRAINING

1. **Funding Source:** Indicate all of the funding source(s) for this study. Check all boxes that apply. Provide information regarding the external funding source. This information should include identification of the agency/sponsor, the funding mechanism (grant or contract), and whether the award is pending or has been awarded. Provide the M/C# and Agency name (if grant-funded). If the funding source associated with a protocol is "pending" at the time of the protocol submission to the HIC (as is the case for most NIH submissions), the PI should note "Pending" in the appropriate section of the protocol application, provide the M/C# and Agency name (if grant-funded) and further note that University (departmental) funds support the research (until such time that an award is made).

PI	Title of Grant	Name of Funding Source	Funding	Funding Mechanism
Federico Vaca, M.D.	Automated Bilingual Computerized Alcohol Screening & Intervention in Latinos.		☒ Federal<br>☐ State<br>☐ Non Profit<br>☐ Industry<br>☐ Other For Profit<br>☐ Other	☐Grant-M#<br>☐Contract#<br>☐Contract Pending<br>☐ Investigator/Department Initiated<br>☐ Sponsor Initiated<br>☐ Other, Specify:
			☐ Federal<br>☐ State<br>☐ Non Profit<br>☐ Industry<br>☐ Other For Profit<br>☐ Other	☐Grant-M#<br>☐Contract#<br>☐Contract Pending<br>☐ Investigator/Department Initiated<br>☐ Sponsor Initiated<br>☐ Other, Specify:

			☐ Federal<br>☐ State<br>☐ Non Profit<br>☐ Industry<br>☐ Other For Profit<br>☐ Other	☐Grant-M#<br>☐Contract#<br>☐Contract Pending<br>☐ Investigator/Department Initiated<br>☐ Sponsor Initiated<br>☐ Other, Specify:

IRB Review fees are charged for projects funded by Industry or Other For-Profit Sponsors. Provide the Name and Address of the Sponsor Representative to whom the invoice should be sent. ***Note: the PI's home department will be billed if this information is not provided.***

Send IRB Review Fee Invoice To:

Name:
Company:
Address:

2. **Research Team:** List all members of the research team. Indicate under the affiliation column whether the investigators or study personnel are part of the Yale faculty or staff, or part of the faculty or staff from a collaborating institution, or are not formally affiliated with any institution. **ALL members of the research team MUST complete Human Subject Protection Training (HSPT) and Health Insurance Portability and Accountability Act (HIPAA) Training before they may be listed on the protocol. See NOTE below.**

NOTE: The HIC will remove from the protocol any personnel who have not completed required training.

	**Name**	**Affiliation: Yale/Other Institution (Identify)**	**NetID**
**Principal Investigator**	Federico Vaca, M.D.	Yale Medical School	
**Role: Co-Investigator**	Gail D'Onofrio, M.D.	Yale Medical School	
**Role: Co-Investigator**	James Dziura, Ph.D.	Yale Medical School	
**Role: Co-Investigator**	Allen Hsiao, M.D.	Yale Medical School	
**Role: Co-Investigator**	Michael Pantalon, Ph.D.	Yale Medical School	
**Role: Correspondent**	Alexei Nelayev	Yale Medical School	

A personnel protocol amendment will need to be submitted when training is completed.

SECTION IV: PRINCIPAL INVESTIGATOR/FACULTY ADVISOR/ DEPARTMENT CHAIR AGREEMENT

As the **principal investigator** of this research project, I certify that:

- The information provided in this application is complete and accurate.
- I assume full responsibility for the protection of human subjects and the proper conduct of the research.
- Subject safety will be of paramount concern, and every effort will be made to protect subjects' rights and welfare.
- The research will be performed according to ethical principles and in compliance with all federal, state and local laws, as well as institutional regulations and policies regarding the protection of human subjects.
- All members of the research team will be kept apprised of research goals.
- I will obtain approval for this research study and any subsequent revisions prior to my initiating the study or any change and I will obtain continuing approval of this study prior to the expiration date of any approval period.
- I will report to the HIC any serious injuries and/or other unanticipated problems involving risk to participants.
- I am in compliance with the requirements set by the University and qualify to serve as the principal investigator of this project or have acquired the appropriate approval from the Dean's Office or Office of the Provost, or the Human Subject Protection Administrator at Yale-New Haven Hospital, or have a faculty advisor.
- I will identify a qualified successor should I cease my role as principal investigator and facilitate a smooth transfer of investigator responsibilities.

_Federico Vaca, M.D.____ June 23, 2014_______
PI Name (PRINT) and Signature Date

As the **faculty advisor** of this research project, I certify that:

- The information provided in this application is complete and accurate.
- This project has scientific value and merit and that the student or trainee investigator has the necessary resources to complete the project and achieve the aims.
- I will train the student investigator in matters of appropriate research compliance, protection of human subjects and proper conduct of research.
- The research will be performed according to ethical principles and in compliance with all federal, state and local laws, as well as institutional regulations and policies regarding the protection of human subjects.
- The student investigator will obtain approval for this research study and any subsequent revisions Prior to initiating the study or revision and will obtain continuing approval prior to the expiration of any approval period.
- The student investigator will report to the HIC any serious injuries and/or other unanticipated problems involving risk to participants.
- I am in compliance with the requirements set forth by the University and qualify to serve as the faculty advisor of this project.

Department Chair's Assurance Statement

Do you know of any real or apparent institutional conflict of interest (e.g., Yale ownership of a sponsoring company, patents, licensure) associated with this research project?

☐ Yes (provide a description of that interest in a separate letter addressed to the HIC.)
☐ No

As Chair, do you have any real or apparent protocol-specific conflict of interest between yourself and the sponsor of the research project, or its competitor or any interest in any intervention and/or method tested in the project that might compromise this research project?

☐ Yes (provide a description of that interest in a separate letter addressed to the HIC)
☐ No

I assure the HIC that the principal investigator and all members of the research team are qualified by education, training, licensure and/or experience to assume participation in the conduct of this research trial. I also assure that the principal investigator has departmental support and sufficient resources to conduct this trial appropriately.

____ _______________
Chair Name (PRINT) and Signature Date

____
Department

YNHH Human Subjects Protection Administrator Assurance Statement

Required when the study is conducted solely at YNHH by YNHH health care providers.

As Human Subject Protection Administrator (HSPA) for YNHH, I certify that:

- I have read a copy of the protocol and approve it being conducted at YNHH.
- I agree to notify the IRB if I am aware of any real or apparent institutional conflict of interest.
- The principal investigator of this study is qualified to serve as P.I. and has the support of the hospital for this research project.

____ ____________________
YNHH HSPA Name (PRINT) and Signature Date

For HIC Use Only

______________________ **Date Approved**	______________________ **Human Investigation Committee Signature**

This protocol is valid through ______________________

SECTION V: RESEARCH PLAN

1. **Statement of Purpose:** State the scientific aim(s) of the study, or the hypotheses to be tested.

The Specific Aims of the study are:

Aim 1. To compare the efficacy of AB-CASI (Automated Bilingual Computerized Alcohol Screening and Intervention) to SC (standard care) in the reduction of alcohol consumption in unhealthy drinkers.

Hypothesis 1. At 12 months, AB-CASI will be superior to SC in reducing the number of binge drinking episodes and the mean number of weekly drinks over the last 28-days.

Aim 2. To compare the efficacy of AB-CASI to SC in the reduction of alcohol-related negative health behaviors and consequences.
Hypothesis 2. At 12 months, AB-CASI will be superior to SC in reducing alcohol-related negative health behaviors and consequences (episodes of impaired driving, riding with an impaired driver, injuries, arrests, tardiness and days absent from work/school).

Aim 3. To compare the efficacy of AB-CASI to SC in 30-day treatment engagement.

Hypothesis 3. AB-CASI will be superior to SC in increasing 30-day treatment engagement in unhealthy drinkers.
Exploratory Aim. To explore variation of AB-CASI on alcohol consumption, alcohol-related negative health behaviors and consequences and 30-day treatment engagement across Latino subpopulations (Puerto-Rican, Mexican-American, Cuban-American, South/Central American) as well as other potential modifiers (age, birthplace, gender, preferred language, dependence, reason for ED visit, and smoking status).

Expected Outcomes and Future Impact. Our study is firmly and purposively aligned with the National Institute of Alcohol Abuse and Alcoholism's (NIAAA) ***Strategic Plan to Address Health Disparities*** and its commitment to expand research capacity that positively impacts minority populations.[27] Further, it coheres with NIH Director Collins' pledge to clearly understand health disparities and to close existing gaps.[28] Our study will be the first randomized controlled trial of automated bilingual ED-SBIRT for Latino drinkers. These data are greatly needed to understand the capacity for efficaciously and practically expanding ED-SBIRT (Emergency Department Screening, Brief Intervention, and Referral to Treatment) in an automated bilingual health information technology platform. This will move the field of alcohol research forward and promote meaningful reductions in existing alcohol-related health disparity gaps.

2. **Background:** Describe the background information that led to the plan for this project. Provide references to support the expectation of obtaining useful scientific data.

Magnitude of the Problem: The burden of disease and toll on human life due to alcohol is staggering. As the most commonly used drug in the U.S., consequences of misuse persist as major short-term and life-long threats to individual and community health.[29,30] Alcohol-related deaths are the 3rd leading cause of preventable death for Americans.[31] Nearly 80,000 alcohol-attributable deaths per year accounted for 2.3 million years of potential life lost from 2000-2005.[32,33] Similarly, the annual financial burden that society bears attributed to alcohol misuse is estimated to be $223.5 billion or $746 per person in the U.S.[34] The cost of alcohol misuse exceeds that of asthma, diabetes and high blood pressure combined.[35] Risky drinkers account for 60% of alcohol-related absenteeism, tardiness, and poor work quality.[36] They utilize twice the health care resources of healthy employees. Alcohol is consistently implicated as a major risk factor for nearly all categories of unintentional (falls, crashes, burns) and intentional (homicide, suicide, assaults, child maltreatment)[37] non-fatal and fatal injury.[30,38,39] It is also a major factor in a multitude of serious chronic medical conditions including gastrointestinal, cardiovascular, mental health disorders, and several terminal cancers.[40,41]

U.S. Latinos and Alcohol, Growing Vulnerability and Disparities: In the last decade, the U.S. Latino population grew by 43% compared to 5% of the non-Latino population to comprise more than 50 million (33.3 million are 18 years and older).[42,43] This increase exceeded half of the U.S. population growth. The average age of the U.S. Latino population is 10 years younger than the overall U.S. population.[44] This population characteristic touches only the very surface of this groups' vulnerability. The size of the U.S. Latino population is expected to triple by 2050. With ongoing national efforts to eliminate health disparities,[45] the rapid population growth of U.S. Latinos has major implications for the extent to which alcohol-related disparities can be reduced. This is of particular concern given the context of the U.S. Latino population growth outpacing any evidence of reduction of existing disparity gaps[46] and the comparatively small number of alcohol-related randomized control trials that have included U.S. Spanish speaking Latinos.

For U.S. Latinos, consequences of drinking[1] and disparities in specialized treatment[47] are profound with a predictable likelihood to worsen as the population growth accelerates. Studies document the greater burden of disease from both social and health perspectives in Latino drinkers as compared to those of other race and ethnicities.[2,48-51] The first National Alcohol Survey to emphasize race and ethnicity was conducted in 1984.[52] This and subsequent studies show the importance of racial and ethnic variations in alcohol consumption and related consequences.[8,53,54] **Latino men have the highest prevalence of daily heavy drinking**[55] **with prolonged duration of heavy drinking**[56] **and are more likely to binge drink.**[29,57] Moreover, although non-Latino Whites are more likely than Latinos to succumb to alcohol dependency in their lifetime[58], **once alcohol dependence occurs, Latinos have a higher prevalence than non-Latino Whites of recurrent or persistent alcohol dependence.**[56,59] **Latinos also have higher rates of alcohol-related consequences, such as driving while intoxicated**[60,61] **and lifetime arrests for driving under the influence of alcohol.**[61] Finally, a paucity of research has focused on the important distinction between ethnic variation as it relates to Latino subgroups (i.e. of Mexican, Cuban, Puerto Rican descent) and alcohol use disorders (AUD).[62-64] While a recent national study shows no significant relationship between acculturation and alcohol-related social

problems, birth place for both Latino men and women is noted as an independent risk factor.[65] **Nearly 50% of U.S. Latinos are foreign born**,[66] and for some Latino subgroups there exists an even greater level of vulnerability within an already "at-risk" population in the context of AUDs. **Herein lies an important opportunity for testing the identification, early intervention, and treatment initiation strategies in this vulnerable population group**.

U.S. Emergency Departments and Alcohol Use Disorders: In 2009, U.S. emergency departments had more than 136 million visits (45.1 ED visits per 100 persons).[5] This was a growth in annual visits of 10% from 2008 and 16.5% from 2007.[5,67,68] **Concurrently, injury visits and ED population diversity have continued to increase with an additional 3 million more ED visits by Latinos in 2009 compared to 2007**.[67-69] Studies show that a large proportion of ED visits are attributed to alcohol.[70-72] Those with AUDs average nearly twice as many injury-related events per year as non-problem drinkers and four times as many hospitalizations for injury.[73] Every day emergency physicians treat patients that present to the ED as a result of unhealthy drinking ranging from at-risk to dependence.[74] [75] At-risk drinking is defined as a quantity or pattern of alcohol use that places someone at-risk for adverse health events. Patients may progress in severity when alcohol use results in adverse events, such as physical or psychological harm.[76,77] **The NIAAA defines drinking "at-risk" for illness or injury as those drinking above low-risk limits (i.e. men > 14 drinks/week or > 4 drinks/occasion; women and all > 65 y/o > 7 drinks/week or > 3 drinks/occasion)**.[78] Compared to dependent drinkers, which represent 5% of the U.S. population, those drinking above the low-risk limits comprise 20%.[79] An early study showed that 17% of all patients presenting to an ED were harmful drinkers.[80] Patients presenting to the ED are not only more likely to have AUDs than those presenting to primary care[6,7] but also 1.5 times more likely to report heavy drinking, experience consequences of drinking, or to have received treatment for an AUD.[81] It is noteworthy that **a single alcohol-related ED visit has been shown to be an important predictor of ongoing problem drinking, alcohol-impaired driving, and premature death.**[82]

EDs Offer an Important Context to Reach Latino Drinkers: Those with AUDs frequently have high rates of medical co-morbidities and are more likely to present to healthcare systems than any other service system.[83] Therefore, alcohol screening and brief intervention in the medical setting is critical.[84-87] Over 19 million Latinos accessed healthcare through U.S. EDs in 2009.[69] With the millions of annual alcohol-related injuries and medical co-morbidity visits to U.S. EDs, these encounters provide an opportunity to identify, intervene, and initiate treatment in the lives of those with AUDs.[14] Over two decades ago, emergency medicine clinicians and researchers recognized the need to systematically identify and intervene in unhealthy drinking ED populations.[37] Many scientifically rigorous ED-SBIRT clinical trials empirically demonstrate reductions in drinking and harm.[12,13,18,22,88-90] **Most recently, an NIAAA funded large randomized ED-SBIRT clinical trial by D'Onofrio et al.**[15] **showed that the use of brief interventions by emergency practitioners in the ED setting improves patient outcomes in English speaking patients by decreasing 7-day alcohol consumption, episodes of binge drinking, and impaired driving.**

Surmounting ED-SBIRT Barriers with Health Information Technology: Today, conflicting demands and increasing responsibilities are the norm for emergency physicians working in busy EDs that frequently exceed their clinical care capacity. Therefore, **brief and effective screening**

APPROVED BY THE YALE UNIVERSITY IRB 3/23/2020

and intervention strategies are a prerequisite "must" if they are to be successfully integrated into the ED visit. Evidence suggests that acute subcritical injury may be an important motivator to reduce drinking.[91] Therefore, an ED visit provides a valuable teachable moment. Unfortunately, despite high rates of heavy drinking among both injured and non-injured ED patients, ED-SBIRT is rarely performed as routine practice. EDs miss critical opportunities to address AUDs and ED-SBIRT practice lags behind national guidelines.[19]

The use of health information technology to facilitate SBIRT outside the ED has been successful.[92,93] It's accuracy is comparable to paper and pencil versions when used with hospital admitted patients.[94] In some clinical settings, evidence supports the efficacy of computerized and web-based motivational interventions for smoking cessation in adults[95] and alcohol/substance abuse prevention in young adults.[96-102] More recently, studies have used computer technology in ED settings to evaluate SBIRT feasibility with compelling outcomes.[12,20,22,23,103,104] In particular, Neumann et al. showed that a German (mono-lingual) computerized ED-SBIRT intervention could yield a significant effect when compared to controls in reducing the proportion of at-risk drinkers at 6 months as well as significant reductions in alcohol consumption at both 6- and 12-months.[12]

Experts promote health information technology to overcome important and persistent barriers encountered in ED-SBIRT implementation.[21,105] **Some of these barriers include practitioner time burden, limited knowledge of brief intervention strategies (that can compromise fidelity), limited resources in screening and intervention personnel, and inability to easily provide ED-SBIRT in multiple languages.**[74,106,107] **The latter limitation severely prevents the reach of ED-SBIRT to some of the most vulnerable populations in the ED.**

Automating ED-SBIRT can eliminate time and resource challenges while providing patient anonymity that lends itself well to self-disclosure of sensitive or taboo subjects. It can optimize intervention fidelity and integrity which is important given the variations that exist in accuracy and reliability of measures across different race and ethnic groups.[21,80,108-112] Added-value in the use of automated ED-SBIRT includes enhanced privacy, reduced social desirability bias, accommodation for patients with poor literacy (providing text and audiographical interface vs. text only), and the ability to write algorithms to automate tailored feed-back and treatment service referral. Moreover, computer touch screen with text-audio interfaces addresses literacy issues and makes automated ED-SBIRT an option for inexperienced computer users. Language translation (text and audio) of the automated ED-SBIRT is the single most important cultural adaptation that can be undertaken. Without it, non-English speaking language groups, often with the greatest need of screening and intervention, are overlooked. Field et al.[16] were successful in demonstrating that ED-SBIRT was efficacious in Latino Spanish speaking patients **with only linguistic translation**. However, this study required extensively trained bilingual providers **(bilingual master's level or degreed clinicians)** who performed the brief motivational intervention. The practicality of implementing this intervention in EDs across the country is seriously limited given the scarcity of bilingual clinicians in addition to other listed barriers. **The use of an automated bilingual ED-SBIRT has already shown strong evidence of feasibility, bilingual ED patient acceptability, and compelling suggestion of efficacy**.[23,26] It is practical and can lead to ED cost savings in several ED-SBIRT areas (personnel, interpreter services, patient recidivism).

This Study Will Improve Knowledge and Advance the Field if Aims are Achieved: The feasibility of developing and implementing automated bilingual ED-SBIRT in a busy ED clinical setting has been demonstrated. Rigorously testing of its efficacy is the next logical step. If automated bilingual ED-SBIRT is shown to be effective, broader dissemination and adoption will occur with the benefits of reduction in intervention cost and practitioner time burden as well as enhanced intervention integrity and fidelity. This will improve the ability of U.S. EDs to reach and provide tailored interventions to vulnerable ED (English and Spanish speaking) groups. It will further improve adherence to existing national SBIRT guidelines that will broaden the base for alcohol use disorder identification and treatment initiation while closing health disparity gaps.

3. **Research Plan:** Summarize the study design and research procedures using non-technical language that can be readily understood by someone outside the discipline. Be sure to distinguish between standard of care vs. research procedures when applicable, and include any flowcharts of visits specifying their individual times and lengths. Describe the setting in which the research will take place.

Our research team will rigorously test the efficacy of AB-CASI[23], against a standard condition (SC) in a first-of-a-kind ED randomized controlled trial. Studying an urban adult (≥ 18 y/o) Latino ED population, the objectives are to demonstrate the superiority of AB-CASI compared to SC in: 1) reduction of alcohol consumption (Aims 1), 2) reduction of negative health behaviors and consequences (Aim 2), and 3) increasing 30-day treatment engagement (Aim 3). We will also explore variation of the AB-CASI intervention on alcohol consumption, alcohol-related negative health behaviors and consequences, and 30-day treatment engagement by Latino subpopulations (Puerto-Rican, Mexican-American, Cuban-American, South/Central American) as well as other potential modifiers (age, birthplace, gender, preferred language, reason for ED visit, and smoking status). English and Spanish speaking Latino adults presenting to the Bridgeport Hospital ED will be screened using the study inclusion and exclusion criteria.

After obtaining voluntary informed written consent, a total of 850 unhealthy drinkers meeting eligibility criteria will be enrolled and randomized. Randomization will be stratified by preferred language (English or Spanish) and dependence status (Yes or No). Equal numbers of preferred English and Spanish speaking participants will be enrolled to permit the exploration of treatment modification by language. In order to ensure the enrollment of consistent drinkers, an initial rapid Health Quiz, inclusive of a 7-day timeline follow-back of alcohol use, will be administered to prospective enrollees. For those found to be in the spectrum of unhealthy drinkers, demographics, preferred language (proxy for acculturation), locator information and baseline alcohol consumption will be collected at enrollment and prior to randomization.

By face-to-face interview (English or Spanish), the AB-CASI and SC groups will undergo **5 brief baseline assessments** (Alcohol Use Disorders Identification Test (AUDIT), Brief Event Data (BED), Revised Injury Behavior Checklist (RIBC), Short Inventory of Problems (SIP), Timeline Follow-back (TLFB)). All unhealthy drinkers randomized to the AB-CASI group will undergo an iPad®-based bilingual automated alcohol screening (via embedded AUDIT) and BNI session. Both study groups of interest will be informed that they will be contacted by telephone

at 30-days, 6-, and 12-months for follow-up assessments. At 30-days, both groups will be contacted and undergo a Treatment Services Review (TSR) to determine if treatment engagement has occurred.

At the 6-month follow-up, 3 brief assessments will be administered to both study groups (BED, TLFB, TSR). **At 12-months, 5 brief assessments** will be administered (BED, SIP, TLFB, RIBC, TSR). The primary self-reported alcohol consumption outcome is the number of episodes of binge drinking over the last 28-days. Secondary outcome measures include mean number of drinks, alcohol-related negative behaviors and consequences, and 30 day treatment engagement. Primary data analyses will focus on an intention-to-treat sample. We will use MIXED model repeated measures analysis to test for differences in alcohol consumption at each follow-up. We will conduct similar analyses using secondary outcome measures.

Schedule of Assessments

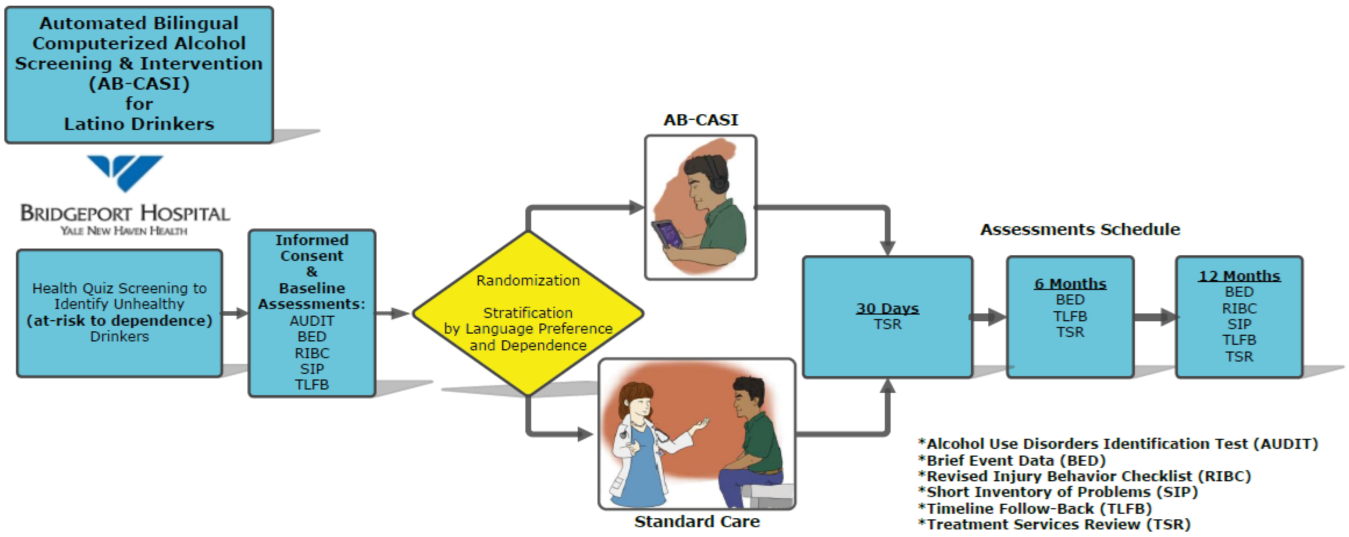

4. Genetic Testing N/A ☒

A. Describe

i. the types of future research to be conducted using the materials, specifying if immortalization of cell lines, whole exome or genome sequencing, genome wide association studies, or animal studies are planned
ii. the plan for the collection of material or the conditions under which material will be received
iii. the types of information about the donor/individual contributors that will be entered into a database
iv. the methods to uphold confidentiality

B. What are the conditions or procedures for sharing of materials and/or distributing for future research projects?
C. Is widespread sharing of materials planned?
D. When and under what conditions will materials be stripped of all identifiers?

E. Can donor-subjects withdraw their materials at any time, and/or withdraw the identifiers that connect them to their materials?
    i. How will requests to withdraw materials be handled (e.g., material no longer identified: that is, anonymized) or material destroyed)?

F. Describe the provisions for protection of participant privacy
G. Describe the methods for the security of storage and sharing of materials

5. **Subject Population:** Provide a detailed description of the types of human subjects who will be recruited into this study.

We will enroll all of our study subjects from Bridgeport Hospital ED. This hospital is located in the city of Bridgeport, Connecticut which has a population of more than 144,000 residents. The US Census notes that within the city of Bridgeport, 44% of households speak another language other than English at home, the proportion of those living in poverty is 20%, and Latinos make up 38% of the city's population. The hospital's primary catchment area encompasses a population of 400,000. The gender, racial, and ethnic characteristics of our proposed urban adult Latino ED population reflects the demographics of Bridgeport City communities.

Inclusion of Women

The Bridgeport Hospital ED population is comprised of 52% women and 48% men. While we will enroll women into our study, no amount of alcohol consumption is known to be safe for a developing fetus. Our study may ultimately end up enrolling female patients found to be drinking above the NIAAA low-risk limits (that meet all other enrollment eligibility criteria) that may not initially be known to be pregnant. If the female patient is randomized to the intervention group (AB-CASI) and she is of child bearing age, she will automatically receive prevention messaging that recommends abstention from alcohol for women of child bearing age that are known to be pregnant or trying to become pregnant. If throughout the course of that study (throughout follow-up), an enrolled female subject is known to become pregnant, she will again receive the appropriate prevention messages and we will encourage her to follow-up with her primary care physician.

Inclusion of Minorities

The proposed study is specifically focused on the enrollment of self-identified Latino adult ED patients. Those that speak either English or Spanish as their primary language, are found to drink above the NIAAA low-risk limits, and meet the other enrollment eligibility criteria will be invited to participate in the study. The annual ED census at Bridgeport Hospital is 77,000 visits. The overall ED population is reflective of the surrounding communities with a diverse racial, ethnicity, and cultural mix. The racial/ethnic makeup of the ED is 35% Latino, 32% White, 31% Black, and Asian/American Indian/Hawaiian Pacific Islander/Other 2%. At least 10% of all the annual visits to the Bridgeport Hospital ED are Spanish speaking patients

6. **Subject classification:** Check off all classifications of subjects that will be specifically recruited for enrollment in the research project. Will subjects who may require additional safeguards or other considerations be enrolled in the study? If so, identify the population of subjects requiring special safeguards and provide a justification for their involvement.

☐ Children	☐ Healthy	☐ Fetal material, placenta, or dead fetus
☐ Non-English Speaking	☐ Prisoners	☒ Economically disadvantaged persons
☐ Decisionally Impaired	☐ Employees	☐ Pregnant women and/or fetuses
☐ Yale Students	☒ Females of childbearing potential	

NOTE: Is this research proposal designed to enroll children who are wards of the state as potential subjects? ☐ Yes ☐ No (If yes, see Instructions section VII #4 for further requirements)

7. **Inclusion/Exclusion Criteria:** What are the criteria used to determine subject inclusion or exclusion?

- **Inclusion criteria:** 1) English and Spanish speaking adult (≥ 18 y/o) Latino ED patients who present to the Bridgeport Hospital's ED and who drink over the NIAAA low-risk limits.

- **Exclusion criteria:** Patients will be excluded for the following reasons: 1) primary language other than English or Spanish; 2) current enrollment in an alcohol or substance abuse treatment program; 4) current ED visit for acute psychosis; 5) condition that precludes interview or AB-CASI use i.e., life threatening injury/illness; 6) in police custody; or 7) inability to provide two contact numbers for follow-up.

- **Vulnerable Populations:** Children (≤18 years old) and prisoners will not be approached for enrollment in the proposed study. While we will enroll women into our study, no amount of alcohol consumption is known to be safe for a developing fetus. Our study may ultimately end up enrolling female patients found to be drinking above the NIAAA low-risk limits (that meet all other enrollment eligibility criteria) that may not initially be known to be pregnant. If the female patient is randomized to the intervention group (AB-CASI) and she is of child bearing age, she will automatically receive prevention messaging that recommends abstention from alcohol for women of child bearing age that are known to be pregnant or trying to become pregnant. If throughout the course of that study (throughout follow-up), an enrolled female subject is known to become pregnant, she will again receive the appropriate prevention messages and we will encourage her to follow-up with her primary care physician. All Federal rules regarding vulnerable subjects will be incorporated into the study protocol, and reviewed by the Yale University Human Investigation Committee (HIC).

8. How will **eligibility** be determined, and by whom?

English and Spanish speaking adult (≥ 18 years old) Latino ED patients at Bridgeport Hospital will be briefly screened to confirm consistent drinking. If they are found to exceed the NIAAA criteria for low-risk drinking from at-risk through dependence, they will be

APPROVED BY THE YALE UNIVERSITY IRB 3/23/2020

offered enrollment into the study. Patients will be excluded from study enrollment for the following reasons:

- primary language other than English or Spanish
- current enrollment in an alcohol or substance abuse treatment program
- current ED visit for acute psychosis
- condition that precludes interview or AB-CASI use i.e., life threatening injury/illness
- in police custody
- inability to provide two contact numbers for follow-up.

Potential subjects will be **evaluated for eligibility by a bilingual trained research associate** that has successfully completed the Yale University Human Investigation Committee-required human subjects protection and HIPAA trainings.

9. **Risks:** Describe the reasonably foreseeable risks, including risks to subject privacy, discomforts, or inconveniences associated with subjects participating in the research.

Our study is of minimal risk to subjects who agree to enroll in the study. While minimal, there is remote risk related to breach of confidentiality. However, all subjects will be reassured that any data collected will be kept strictly confidential. All study personnel are required to successfully complete human research protection and Health Insurance Portability and Accountability Act (HIPAA) training prior to working with any subject data. All study subjects will be assured that if they should choose not to participate in the proposed study, their decision will in no way affect their medical care. Further, at no time will study procedures be allowed to interfere with any of the ED medical care of the patient. The alternative to participating in this study is to choose not to participate.

10. **Minimizing Risks:** Describe the manner in which the above-mentioned risks will be minimized.

The main risk associated with the study is the possibility that confidential information obtained during the study will be disclosed. All consented and enrolled patients will be assigned a patient identification number (PID) that will be used on any data collection forms and AB-CASI assessment. All patient interactions will be conducted in areas that are as private as possible; typically private or semi-private ED rooms. All study subjects will be assured that should they choose not to participate in the proposed study, their decision will in no way affect their medical care. Moreover, we will assure these ED patients that their decision not to participate in the proposed study will not be known to anyone except the research personnel and once again reiterate that this decision will not affect their medical care. Finally, at no time will study procedures be allowed to interfere with any of the ED medical care of the patient.

Only authorized members of the research team will have access to patient refusal, consent, and/or enrollment information. Any patient identifiable information that is obtained in connection with this study will remain confidential and will be disclosed only with subject permission or as required by U.S. federal or State law.
All electronic subject data will be password protected and kept on a password protected secure server. Data will only be reported in aggregate. Data will be de-identified prior to

APPROVED BY THE YALE UNIVERSITY IRB 3/23/2020

formal data analysis. Upon completion of the study, all digital subject datasets will be stored in a password-protected study computer, to which only the PI, authorized co-investigators and study personnel will have access. All hardcopy study files with identifying subject information will remain in locked file cabinets in the locked office of the PI until they are destroyed and after all analyses are complete. This protocol presents minimal risks to the consented and enrolled subjects and adverse events or other problems are not anticipated. However, in the unlikely event that such events occur, they will be reported in writing within 48 hours to the Yale University Human Investigation Committee and all appropriate funding and regulatory agencies. The principal investigator will apprise fellow investigators and authorized study personnel of all adverse events that occur during the conduct of this research project through regular study meetings or via email as they are reviewed by the PI.

Children (≤18 years old) and prisoners will not be approached for enrollment in the propose study. While we will enroll women into our study, no amount of alcohol consumption is known to be safe for a developing fetus. Our study may ultimately end up enrolling female patients found to be drinking above the NIAAA low-risk limits (that meet all other enrollment eligibility criteria) that may not initially be known to be pregnant. If the female patient is randomized to the intervention group (AB-CASI) and she is of child bearing age, she will automatically receive prevention messaging that recommends abstention from alcohol for women of child bearing age that are known to be pregnant or trying to become pregnant. If throughout the course of that study (throughout follow-up), an enrolled female subject is known to become pregnant, she will again receive the appropriate prevention messages and we will encourage her to follow-up with her primary care physician. All Federal rules regarding vulnerable subjects will be incorporated into the study protocol, and reviewed by the Yale University Human Investigation Committee (HIC). Confidentiality of collected patient data will be maintained with the use of a study patient identification reference number system maintained by the study investigators. The name of consented and enrolled subjects will appear only on a consent form and "key" form kept in a secure and locked cabinet by the PI and separate from the enrolled subject files. All collected materials will be kept in locked file cabinets in the locked office of the principal investigator. We also plan to obtain a Confidentiality Certificate from the Department of Health and Human Services (DHHS) to ensure the confidentiality of all records and data.

11. **Data and Safety Monitoring Plan:** Include an appropriate Data and Safety Monitoring Plan (DSMP) based on the investigator's risk assessment stated below. (Note: the HIC will make the final determination of the risk to subjects.) For more information, see the Instructions, page 24.
    a. What is the investigator's assessment of the overall risk level for subjects participating in this study?

        The proposed study is a minimal risk study. Consistent with the Data and Safety Monitoring Plan (DSMP) model of the Yale University School of Medicine and the Bridgeport Hospital (Yale-New Haven Health), the DSMP includes provisions for data review and performance of safety reviews, as described below.

APPROVED BY THE YALE UNIVERSITY IRB 3/23/2020

b. If children are involved, what is the investigator's assessment of the overall risk level for the children participating in this study?

   Children are not involved.

c. Copy, paste, and then tailor an appropriate Data and Safety Monitoring Plan from http://www.yale.edu/hrpp/forms-templates/biomedical.html for
   i. Minimal risk

The PI and co-investigators will meet on a monthly basis to discuss study progress and any adverse events. The data safety monitoring board (DSMB) will consist of the PI and co-investigators. The DSMB will meet every six months to review interim data analyses and any adverse events as needed. In addition, the Yale University Human Investigation Committee and the Bridgeport Hospital Institutional Review Board will review the study protocol at least once a year, and will review as well as approve any amendments before they are made to the protocol. Any adverse effects encountered will be reported promptly to the Yale University Human Investigation Committee, the Bridgeport Hospital Institutional Review Board, and to the funding agency, according to current regulations.

Data and safety monitoring procedures in this study include computer (electronic) data collection and monitoring systems and an organizational structure of clearly defined tasks assigned to all research and clinical personnel involved in the conduct of this study. The computerized study tracking system consists of a database system that records research activities including randomization and follow-up activities. The project director will use this database to monitor ongoing enrolled subject participation. Research associates will digitize collected study data using specialized data entry software (e.g., SPSS). This will facilitate efficient data entry and allow for elimination of out-of-range values and double entry of data, which is likely the result of key punch errors. All error corrections will be fully documented in the research records of the study. All research personnel are required to successfully complete the Yale University Human Investigation Committee and the Bridgeport Hospital Institutional Review Board initial and ongoing training in protection of human subjects and the responsible conduct of scientific research.

All adverse events are reported using the Yale University Human Investigation Committee standard template for reporting adverse events. All adverse events are also reported to the Bridgeport Institutional Review Board. The PI reviews all adverse events, classifies the attribution of adverse events (e.g., definitely, probably, possibly related; unlikely or unrelated) and grades the severity of the event, utilizing the FDA's definition of serious adverse events, on a 6-point scale (0=no adverse event or within normal limit; 1=mild; 2=moderate; 3=severe; 4=life-threatening; 5=fatal). Serious unanticipated or anticipated adverse events will be reported immediately to the Yale University Human Investigation Committee, the Bridgeport Hospital Institutional Review Board, and to the NIAAA. Adverse events will be reported in summary form at least annually to the Yale University Human Investigation Committee and the Bridgeport Hospital Institutional Review Board. The summary will include the number of subjects enrolled and a summary

of graded adverse events to date, using the chart format included in the Yale University DSMP template. The PI will evaluate all adverse events and determine whether the event affects the Risk/Benefit ratio of the study and whether modifications to the protocol (e.g., Risks to Subjects) or consent form (e.g., Risks and Inconveniences) are required.

d. For multi-site studies for which the Yale PI serves as the lead investigator: This is not a multi-site study.

i. How will adverse events and unanticipated problems involving risks to subjects or others be reported, reviewed and managed?
ii. What provisions are in place for management of interim results?
iii. What will the multi-site process be for protocol modifications?

12. **Statistical Considerations:** Describe the statistical analyses that support the study design.

- **General considerations:** This is a randomized, controlled, parallel group trial to evaluate the efficacy of AB-CASI compared to SC to reduce alcohol consumption in adult (≥ 18 y/o) Latino unhealthy drinkers identified in the ED. Data analysis will be conducted in collaboration with James Dziura, PhD and the Yale Center for Analytic Sciences. For all analyses, two-sided significance tests will be implemented and will be performed using SAS v9.2 (SAS Institute, Cary, NC).

- **Justification of Sample Size:** Estimation of sample size is based on randomizing and following a sufficient number of unhealthy drinkers to evaluate the primary hypothesis (Aim 1). Power calculations are provided for Aims 2 and 3 based on the sample size determined for Aim 1. The primary hypothesis is that AB-CASI will result in greater 12-month reductions in the primary outcome, the number of episodes of binge drinking over the past 28-days, which is assessed using the 28-day timeline follow-back method, when compared to SC. Fleming et al.[132] demonstrated that the number of binge episodes in the past 30-days was reduced by 1.14 in the intervention compared to control conditions. D'Onofrio et al.[15] reported similar findings in an RCT conducted in hazardous and harmful drinkers. Given the following: 1) power of 80%, 2) a two-sided 0.05 significance level, 3) a standard deviation for number of binge episodes in the past 28 days of 5.2, and 4) a 1:1 intervention allocation, a sample size of 327 subjects per group will be required to detect a 1.14 difference between AB-CASI and SC in the number of binge episodes in the past 28 days at 12 months. A total of 850 unhealthy drinkers will be enrolled and randomized to accommodate up to 20% dropout. To maximize the ability to explore modification by preferred language, we will enroll an equal number of preferred English (n=410) and Spanish (n=410) speaking Latinos.

  Comparison of secondary outcomes between study groups will be evaluated at the two-sided 0.01 significance level to control inflated type I error from multiple significance testing. Our previous study[123,124] of at-risk individuals demonstrated a difference between subjects receiving a BNI compared to SC of 2.2 drinks per week at 12 months with a standard deviation of 7.5. In their review of brief behavioral counseling in primary care settings, Whitlock et al.[137] found that good quality brief intervention trials reduced consumption by 2.9 to 8.7 drinks per week. Similar conclusions were determined in a meta-analysis by Kaner et al.[138] The estimated sample size of 327 per group will provide 80% power to detect

APPROVED BY THE YALE UNIVERSITY IRB 3/23/2020

differences between intervention groups of 2.0 in the number of drinks per week. In general for continuous outcomes such as the SIP, the sample size above will allow for the detection of small effect sizes (d=0.27) with 80% power. For dichotomous secondary outcomes such as impaired driving, riding with an impaired driver, injuries or arrests, the study will have 80% power to detect absolute risk reductions of 13%, 10% and 7% for SC proportions with events of 50, 20 and 10% respectively.

For Aim 3, allowing for a 15% dropout at 30-days there will be close to 700 subjects that complete their 30-day Treatment Services Review follow-up. With a relatively low rate of expected 30-day treatment engagement in the SC group (conservatively, no greater than 20%), we'll have 80% power at the two-sided 0.01 significance level to detect an increase of 12% in the AB-CASI group.

Confidence in Reaching Target Sample (n=850): The Bridgeport Hospital Emergency Department has 77,000 visits per year of which 82% are adults (n=63,140), 65% are non-repeat visits (41,041), and 35% are Latino (n=14,364). In a 2012 recent survey (n=699), a representative sample was collected during each shift and each day of the week. The survey was offered in both English and Spanish at Bridgeport Hospital. 12.8% of the ED patients reported their language of preference as Spanish meaning 8,082 patients' first language is Spanish or 1/3 of the Bridgeport Latino ED population prefers Spanish. The total number of adult Latino first-time patients expected to present to the Bridgeport Hospital during the 42 month enrollment period is 50,275 or 1,200 per month. Research has found that 60% of Latinos drink and that of those persons, 42% have binged in the past year. Extrapolating this number to the Bridgeport ED population means that 302 Latino first-time ED patients would meet the inclusion criteria per month. To reach our target of 850 study subjects, we only need to enroll 19.5 persons per month for 42 months. Based on surveys in the Bridgeport ED, we estimate that 100 adult Latinos of the 302 Latinos prefer to speak Spanish. Thus, our ability to enroll 19.5 Latinos with half speaking Spanish only per month is reasonable.

- **Data Monitoring:** Procedures for data collection, data management, monitoring of data quality and data analysis have been developed and refined in our previous ED studies. An experienced data manager and the PI will supervise this process. These procedures include use of a computerized database system to monitor clinical and research activities, screening and enrollment, compliance with protocol and treatment interventions, completion of scheduled assessments, and data retrieval. Data quality will be ensured by: 1) extensive training/supervision of research assistants in data collection; 2) preliminary review of all assessment instruments prior to data entry and checks for completeness and coding errors; 3) double data entry of assessment instruments using specialized data entry software (Microsoft Access); 4) error-checking statistical programs. No interim looks for efficacy are planned.

- **Baseline comparability:** Because of the size of this study, we expect that the randomization process will produce reasonably comparable groups. However, the adequacy of the randomization will be assessed by comparing the distribution of baseline demographic and clinical characteristics among the intervention groups. Comparability for continuous variables will be examined graphically and by summary statistics (means, medians, quartiles, etc.). Categorical variables will be examined by calculating frequency distributions. If baseline

differences are observed, the impact of these differences will be evaluated through covariate adjustment of the models described below.

- **Analysis for Aim 1:** The primary objective of the analysis is to demonstrate that AB-CASI will reducealcohol consumption more than SC in unhealthy drinkers at 12 months. The primary outcome (number of binge episodes in past 28-days) will be assessed using the 28-day timeline follow-back method at baseline (prior to initiation of intervention), 6- and 12-months after the intervention initiation. Likelihood-based ignorable analysis using a mixed model will be used to compare alcohol consumption between groups.[139,140] The primary advantage of the mixed model when compared to commonly used methods such as complete case analysis and single imputation (e.g. last observation carried forward) is its flexibility in handling missing data. This analysis will assume that missing data occurs at random (i.e. MAR, not informative). The inclusion of baseline 6- and 12-months outcome data in the model will assist in meeting this assumption. Furthermore, we will evaluate patterns of missing data as well as determine baseline characteristics that are predictive of dropout. If identified, these characteristics will be included in the model to meet the MAR assumption. A generalized linear mixed model (GLMM) with a Negative Binomial distribution will be used to estimate differences in the number of binge drinking episodes in the past 28 days. More specifically the mixed model will include fixed effects for intervention (AB-CASI vs. SC), time (6, 12 months), and the interaction of intervention with time. Additional fixed effects will be included for baseline covariates (baseline number of drinks per week, baseline number of binge episodes, gender, English vs. Spanish preferred language and dependence status). Modification of the intervention effect by preferred language will be evaluated at the 0.10 significance level by including two and three-way interactions of language with intervention and time. If not significant, these interactions will be excluded and intervention effects pooled across preferred language strata. Similar procedures will be used to assess modification by dependence status. Linear contrasts (at the 0.05 two-sided significance level) will be used to estimate intervention group differences and 95% confidence intervals at the 6- and 12-month time points. Using a linear mixed effects model, a similar analysis will be performed for the secondary outcome mean number of drinks per week over the last 28-days.

- **Analysis for Aim 2:** The primary objective of Aim 2 is to demonstrate that negative behaviors and consequences (episodes of impaired driving, riding with an impaired driver, injuries, arrests, tardiness, days absent from work/school and SIP) during the 12-month follow-up will be improved in subjects receiving AB-CASI compared to those receiving SC. A similar repeated measures mixed model analysis as that specified for Aim 1 will be implemented for each of the deleterious outcomes assessed using the Brief Event Data. In addition, outcomes assessed at only 12-months will be evaluated using analysis of covariance with fixed effects for intervention and preferred language strata and baseline outcome as a covariate. Comparison of all secondary outcomes between study groups will be evaluated at the two-sided 0.01 significance level to control inflated type I error from multiple significance testing.

- **Analysis for Aim 3:** The objective of Aim 3 is to determine the effect of the AB-CASI compared to SC on 30-day treatment engagement. We hypothesize that AB-CASI will be superior to SC. Mantel-Haenszel chi-square analysis will be used to compare the likelihood

of 30-day treatment engagement in AB-CASI to SC while adjusting for preferred language and dependence status. Significance will be judged at the two-sided 0.05 significance level. Heterogeneity of treatment effect will be evaluated by the Breslow-Day test. Participants dropping out or lost-to-follow-up will be considered to be not engaged in treatment for the primary analysis.

- **Analyses for the Exploratory Aim:** The objective of the exploratory aim is to determine whether specific factors assessed at baseline (Latino ancestry, age, birthplace, gender, preferred language, reason for ED visit and smoking status) modify the effect of the AB-CASI intervention on alcohol consumption, negative behaviors and consequences as well as 30-day treatment engagement rates. These subgroup analyses will be conducted within the Generalized Linear Mixed Model framework in an evaluation similar to that proposed for investigating modification by the stratification factors of dependence status and preferred language as described above (see Aim 1). Significant interactions will be followed by the estimation and summarization of intervention effects within subgroups at both 6- and 12-month time points.

- **Plan for Missing Data:** Several strategies will be imposed to accommodate the likelihood that missing data will occur during this study. Prevention is the most obvious and effective manner to control bias and loss of power from missing data.[141] This protocol will follow the intent to treat principle, requiring follow-up of all subjects randomized regardless of the actual treatment received.[142] Telephone visit reminders will be delivered to participants prior to protocol specified collection times. Alternative contact information will be identified on entry into the study to minimize loss-to follow-up. Timely data entry combined with weekly missing data reports will trigger protocols for tracking and obtaining missing data items or outcome assessments. Despite these prevention efforts it is reasonable to assume missing data will occur. Our primary analysis is valid under the assumption that missing data is missing at random (MAR).[140] We will evaluate the plausibility of this assumption by determining the extent of missing data and using logistic regression to identify factors associated with dropout. While we do not expect differential rates of dropout between groups or high loss to follow-up, sensitivity analysis using pattern-mixture and selection models under missing not at random (MNAR) assumptions will be performed to examine the robustness of conclusions of the primary analysis to missing data.[140,141]

SECTION VI: RESEARCH INVOLVING DRUGS, BIOLOGICS, RADIOTRACERS, PLACEBOS AND DEVICES

If this section (or one of its parts, A or B) is not applicable, state N/A and delete the rest of the section. **N/A.**

SECTION VII: RECRUITMENT/CONSENT AND ASSENT PROCEDURES

1. Targeted Enrollment: Give the number of subjects:

a. targeted for enrollment at Yale for this protocol.

All of the study subjects to be enrolled
in our study will be enrolled at Bridgeport Hospital in Bridgeport, CT.

A total of 850 urban Latino ED patients that are identified as unhealthy drinkers will be enrolled in our study. Subjects will be identified in the Bridgeport Hospital ED for potential enrollment based on the results of a brief Health Quiz screening. If the patient is found to be drinking above the NIAAA low-risk limits and fulfills all of the other study eligibility criteria, they will be approached for written consent and study enrollment. There is no upper age limit proposed for study enrollment and children under 18 years of age will not be asked to participate in the study.

b. If this is a multi-site study, give the total number of subjects targeted across all sites.
N/A/

2. Indicate recruitment methods below. Attach copies of any recruitment materials that will be used.

☐ Flyers	☐ Internet/Web Postings	☐ Radio
☐ Posters	☐ Mass E-mail Solicitation	☐ Telephone
☐ Letter	☐ Departmental/Center Website	☐ Television
☐ Medical Record Review	☐ Departmental/Center Research Boards	☐ Newspaper
☐ Departmental/Center Newsletters	☐ Web-Based Clinical Trial Registries	
☐ YCCI Recruitment database	☐ Clinicaltrials.gov Registry (do not send materials to HIC)	
☒ Other (describe):		

Potential subjects will be evaluated for eligibility by a bilingual trained research associate that has successfully completed the Yale University Human Investigation Committee-required human subjects protection and HIPAA trainings.

3. Recruitment Procedures:

a. Describe how potential subjects will be identified.

English and Spanish speaking adult (≥ 18 years old) Latino ED patients at Bridgeport Hospital will be briefly screened to confirm consistent drinking. If they are found to exceed the NIAAA criteria for low-risk drinking from at-risk through dependence, they will be offered enrollment into the study. Patients will be excluded from study enrollment for the following reasons:

- primary language other than English or Spanish
- current enrollment in an alcohol or substance abuse treatment program
- current ED visit for acute psychosis
- condition that precludes interview or AB-CASI use i.e., life threatening injury/illness
- in police custody
- inability to provide two contact numbers for follow-up.

b. Describe how potential subjects are contacted.

Bilingual research associates (RA) trained in surveillance techniques will collect locator information.[123,124] They will initially interview all patients face-to-face in English or Spanish. Patients will be asked to provide their cellular and home phone numbers, home address, as well as work phone numbers and address for working patients. Patients will also be asked to provide a Facebook address if they have one, so that we can contact them privately via Messenger. Patients will be asked to identify two locators. One locator will be a family member, or close friend not currently living with the patient. The other locator will be a friend or housemate. Patients will be asked, *"If I cannot reach you, who can I call to relay information that I need to speak with you."* In addition, patients will be asked about informal and formal support systems. They will also be asked where they receive their health care, including hospitals, community health clinics, EDs, etc. Information about such support systems may be used when trying to reach the patient for a scheduled follow-up. At enrollment, the 30-day, and the 6-month interview will be scheduled prior to patient's discharge from the ED. Particular emphasis will be given to identifying days and times convenient to the patient. Once consent and locator information is obtained, the brief baseline assessment will be performed followed by random assignment to the AB-CASI or SC condition.

c. Who is recruiting potential subjects?

Potential subjects will be evaluated for eligibility and recruited by a bilingual trained research associate that has successfully completed the Yale University Human Investigation Committee-required human subjects protection and HIPAA trainings.

4. **Screening Procedures**

a. Will email or telephone correspondence be used to screen potential subjects for eligibility prior to the potential subject coming to the research office? ☐ Yes ☒ No

b. If yes, identify below all health information to be collected as part of screening and check off any of the following HIPAA identifiers to be collected and retained by the research team during this screening process.

HEALTH INFORMATION TO BE COLLECTED:

HIPAA identifiers:

☐ Names
☐ All geographic subdivisions smaller than a State, including: street address, city, county, precinct, zip codes and their equivalent geocodes, except for the initial three digits of a zip code if, according to the current publicly-available data from the Bureau of the Census: (1) the geographic unit formed by combining all zip codes with the same three initial digits contains more than 20,000 people, and (2) the initial three digits of a zip code for all such geographic units containing 20,000 or fewer people is changed to 000.
☐ Telephone numbers
☐ Fax numbers
☐ E-mail addresses
☐ Social Security numbers
☐ Medical record numbers
☐ Health plan beneficiary numbers
☐ Account numbers

☐ All elements of dates (except year) for dates related to an individual, including: birth date, admission date, discharge date, date of death, all ages over 89 and all elements of dates (including year) indicative of such age, except that such ages and elements may be aggregated into a single category of age 90 or older
☐ Certificate/license numbers
☐ Vehicle identifiers and serial numbers, including license plate numbers
☐ Device identifiers and serial numbers
☐ Web Universal Resource Locators (URLs)
☐ Internet Protocol (IP) address numbers
☐ Biometric identifiers, including finger and voice prints
☐ Full face photographic images and any comparable images
☐ Any other unique identifying numbers, characteristics, or codes

5. **Assessment of Current Health Provider Relationship for HIPAA Consideration:**
Does the Investigator or any member of the research team have a direct existing clinical relationship with any potential subject?
☐ Yes, all subjects
☐ Yes, some of the subjects
☒ No

If yes, describe the nature of this relationship.

6. **Request for waiver of HIPAA authorization:** (When requesting a waiver of HIPAA Authorization for either the entire study, or for recruitment purposes only. Note: if you are collecting PHI as part of a phone or email screen, you must request a HIPAA waiver for recruitment purposes.)

Choose one: For entire study: ______ For recruitment purposes only: ______

i. Describe why it would be impracticable to obtain the subject's authorization for use/disclosure of this data;
ii. If requesting a waiver of **signed** authorization, describe why it would be impracticable to obtain the subject's signed authorization for use/disclosure of this data;

By signing this protocol application, the investigator assures that the protected health information for which a Waiver of Authorization has been requested will not be reused or disclosed to any person or entity other than those listed in this application, except as required by law, for authorized oversight of this research study, or as specifically approved for use in another study by an IRB.

Researchers are reminded that unauthorized disclosures of PHI to individuals outside of the Yale HIPAA-Covered entity must be accounted for in the "accounting for disclosures log", by subject name, purpose, date, recipients, and a description of information provided. Logs are to be forwarded to the Deputy HIPAA Privacy Officer.

7. **Required HIPAA Authorization:** If the research involves the creation, use or disclosure of protected health information (PHI), separate subject authorization is required under the HIPAA Privacy Rule. Indicate which of the following forms are being provided:
☐ Compound Consent and Authorization form

☐ HIPAA Research Authorization Form

8. **Consent Personnel:** List the names of all members of the research team who will be obtaining consent/assent.

   Federico Vaca, M.D.

9. **Process of Consent/Assent:** Describe the setting and conditions under which consent/assent will be obtained, including parental permission or surrogate permission and the steps taken to ensure subjects' independent decision-making.

   Patients will be recruited from Bridgeport Hospital ED in Bridgeport, Connecticut. Potential subjects will be evaluated for eligibility by a bilingual trained research associate that has successfully completed the Yale University Human Investigation Committee-required human subjects protection and HIPAA trainings. Subjects will be informed that they are free to decline participation and withdraw from the study at any time and that this choice will in no way adversely affect their health care treatment or their relationship their physician, nurse, study or hospital personnel. After fully informing eligible subjects about the study and answering any questions, written informed consent will be obtained using approved Yale Human Investigation Committee consent and privacy documents. Consent to contact specialized alcohol treatment agencies will be part of the original consent. All subjects will be required to provide written consent in order to participate.

10. E**valuation of Subject(s) Capacity to Provide Informed Consent/Assent:** Indicate how the personnel obtaining consent will assess the potential subject's ability and capacity to consent to the research being proposed.

    All subjects will be reassured that any data collected will be kept strictly confidential. All study personnel are required to successfully complete human research protection and Health Insurance Portability and Accountability Act (HIPAA) training prior to working with any subject data. All study subjects will be assured that if they should choose not to participate in the proposed study, their decision will in no way affect their medical care. Further, at no time will study procedures be allowed to interfere with any of the ED medical care of the patient. All subjects will be informed that the alternative to participating in this study is to choose not to participate.

11. **Documentation of Consent/Assent:** Specify the documents that will be used during the consent/assent process. Copies of all documents should be appended to the protocol, in the same format that they will be given to subjects.

    Compound Consent and Authorization form.

12. **Non-English Speaking Subjects:** Explain provisions in place to ensure comprehension for research involving non-English speaking subjects. Translated copies of all consent materials must be submitted for approval prior to use.

Potential subjects will be evaluated for eligibility by a bilingual (English-Spanish) trained research associate. A copy of the English consent will be translated into Spanish.

13. **Consent Waiver: In certain circumstances, the HIC may grant a waiver of signed consent, or a full waiver of consent, depending on the study.** If you will request either a waiver of consent, or a waiver of signed consent for this study, complete the appropriate section below.
    - ☒ **Not Requesting a consent waiver**
    - ☐ **Requesting a waiver of signed consent**
    - ☐ **Requesting a full waiver of consent**

A. Waiver of **signed** consent: (Verbal consent from subjects will be obtained. **If PHI is collected, information in this section must match Section VII, Question 6**)

☐ **Requesting a waiver of signed consent for Recruitment/Screening only**

If requesting a waiver of signed consent, please address the following:

a. Would the signed consent form be the only record linking the subject and the research?
☐ Yes ☐ No

b. Does a breach of confidentiality constitute the principal risk to subjects?
☐ Yes ☐ No

OR

c. Does the research activity pose greater than minimal risk?
☐ Yes ***If you answered yes, stop. A waiver cannot be granted.*** Please note: Recruitment/screening is generally a minimal risk research activity
☐ No

AND

d. Does the research include any activities that would require signed consent in a non-research context? ☐ Yes ☐ No

☐ **Requesting a waiver of signed consent for the Entire Study (**Note that an information sheet may be required.)

If requesting a waiver of signed consent, please address the following:

a. Would the signed consent form be the only record linking the subject and the research?
☐ Yes ☐ No

b. Does a breach of confidentiality constitute the principal risk to subjects?
☐ Yes ☐ No

OR

c. Does the research pose greater than minimal risk? ☐ Yes ***If you answered yes, stop. A waiver cannot be granted.*** ☐ No

AND

d. Does the research include any activities that would require signed consent in a non-research context? ☐ Yes ☐ No

B. **Full waiver** of consent: (No consent from subjects will be obtained for the activity.)

☐ **Requesting a waiver of consent for Recruitment/Screening only**

a. Does the research activity pose greater than minimal risk to subjects?

☐ Yes ***If you answered yes, stop. A waiver cannot be granted.*** Please note: Recruitment/screening is generally a minimal risk research activity

☐ No

b. Will the waiver adversely affect subjects' rights and welfare? ☐ Yes ☐ No

c. Why would the research be impracticable to conduct without the waiver?

d. Where appropriate, how will pertinent information be returned to, or shared with subjects at a later date?

☐ **Requesting a full waiver of consent for the Entire Study (Note: If PHI is collected, information here must match Section VII, question 6.)**

If requesting a full waiver of consent, please address the following:

a. Does the research pose greater than minimal risk to subjects?

☐ Yes ***If you answered yes, stop. A waiver cannot be granted.***

☐ No

b. Will the waiver adversely affect subjects' rights and welfare? ☐ Yes ☐ No

c. Why would the research be impracticable to conduct without the waiver?

d. Where appropriate, how will pertinent information be returned to, or shared with subjects at a later date?

SECTION VIII: PROTECTION OF RESEARCH SUBJECTS

Confidentiality & Security of Data:

a. What protected health information (medical information along with the HIPAA identifiers) about subjects will be collected and used for the research?

We will collect data that is inclusive of age, gender, race, preferred language (also to be used as a proxy for acculturation), educational level, income, and insurance status.

b. How will the research data be collected, recorded and stored?

Most data will be collected initially on paper data collection sheets and later transferred to a secure computer database.

c. How will the digital data be stored? ☐ CD ☐ DVD ☐ Flash Drive ☐ Portable Hard Drive ☒ Secured Server ☐ Laptop Computer ☐ Desktop Computer ☐ Other

d. What methods and procedures will be used to safeguard the confidentiality and security of the identifiable study data and the storage media indicated above during and after the subject's participation in the study?

Data sheets that are collected will be transported to a locked file cabinet. Data will be entered into a password-protected computer database and stored in a locked office with access available only to the PI, co-investigators, and study personnel.

Do all portable devices contain encryption software? ☒ Yes ☐ No
If no, see http://hipaa.yale.edu/guidance/policy.html

e. What will be done with the data when the research is completed? Are there plans to destroy the identifiable data? If yes, describe how, by whom and when identifiers will be destroyed. If no, describe how the data and/or identifiers will be secured.

All paper files with subject information will remain in locked files in the study office of the PI. At the end of data collection, any identifiable data/PHI will be removed from the database. After this is done, only de-identified data will be maintained in a password-protected file on a password-protected network server to which only the PI, co-investigators and study personnel will have access. After publication, all remaining paper and electronic data will be destroyed.

f. Who will have access to the protected health information (such as the research sponsor, the investigator, the research staff, all research monitors, FDA, Yale Cancer Center Data and Safety Monitoring Committee (DSMC), SSC, etc.)? (please distinguish between PHI and de-identified data).

Only the study team listed in the application will have access to the protected health information.

g. If appropriate, has a Certificate of Confidentiality been obtained?

The PI will obtain a Certificate of Confidentiality.

h. Are any of the study procedures likely to yield information subject to mandatory reporting requirements? No. (e.g. HIV testing – reporting of communicable diseases; parent interview - incidents of child abuse, elderly abuse, etc.). Please verify to whom such instances will need to be reported.

SECTION IX: POTENTIAL BENEFITS

Potential Benefits: Identify any benefits that may be reasonably expected to result from the research, either to the subject(s) or to society at large. (Payment of subjects is not considered a benefit in this context of the risk benefit assessment.)

The proposed study is of minimal risk to the subjects. Potential benefits for the enrolled study subjects include the possibility of changing any alcohol use behavior to a more health promoting and injury prevention pattern.

Using the most current knowledge and process of ED-SBIRT and testing the efficacy of a AB-CASI is essential to surmount persistent barriers to ED-SBIRT and expand the reach of ED-

SBIRT to vulnerable ED populations. This minimal risk project will provide can provide short and long-term health outcomes of unhealthy drinkers that present to the ED. The results of this study will move the field alcohol and health disparities research forward. Found efficacy can facilitate greater adoption and implementation of ED-SBIRT programs within EDs throughout the nation.

SECTION X: RESEARCH ALTERNATIVES AND ECONOMIC CONSIDERATIONS

1. **Alternatives:** What other alternatives are available to the study subjects outside of the research?

   The alternative not to participate in the study.

2. **Payments for Participation (Economic Considerations):** Describe any payments that will be made to subjects, the amount and schedule of payments, and the conditions for receiving this compensation.

   All consented and enrolled ED patients subjects will also receive monetary incentive for participating in each of the 4 stages of assessment in the study protocol ($20 @ baseline assessment; $25 @ 30-day follow-up; $40 @ 6-month assessment; $50 @ 12-month assessment). The monetary incentive will be provided in the form of a popular department store gift card.

3. **Costs for Participation (Economic Considerations):** Clearly describe the subject's costs associated with participation in the research, and the interventions or procedures of the study that will be provided at no cost to subjects.

   There are no costs associated with participation in this research.

4. **In Case of Injury:** This section is required for any research involving more than minimal risk.
   a. Will medical treatment be available if research-related injury occurs?
   b. Where and from whom may treatment be obtained?
   c. Are there any limits to the treatment being provided?
   d. Who will pay for this treatment?
   e. How will the medical treatment be accessed by subjects?

Bibliography & References Cited

1. Keyes KM, Liu XC, Cerda M. The role of race/ethnicity in alcohol-attributable injury in the United States. *Epidemiologic reviews.* Jan 2012;34(1):89-102.
2. Mulia N, Ye Y, Greenfield TK, Zemore SE. Disparities in alcohol-related problems among white, black, and Hispanic Americans. *Alcoholism, clinical and experimental research.* Apr 2009;33(4):654-662.
3. Chartier K, Caetano R. Ethnicity and Health Disparities in Alcohol Research. *Alcohol research & health : the journal of the National Institute on Alcohol Abuse and Alcoholism.* 2010;33(1-2):152-160.
4. Ennis SR, Ríos-Vargas M, Albert NG. *The Hispanic Population: 2010 Census Brief.* U.S. Census Bureau;2011. C2010BR-04.
5. Center for Disease Control and Prevention. *National Hospital Ambulatory Medical Care Survey: Fact Sheet Emergency Department* U.S. Department of Health and Human Services;2011.
6. Bernstein E, Bernstein J, Levenson S. Project ASSERT: an ED-based intervention to increase access to primary care, preventive services, and the substance abuse treatment system. *Ann Emerg Med.* Aug 1997;30(2):181-189.
7. Cherpitel CJ. Emergency room and primary care services utilization and associated alcohol and drug use in the United States general population. *Alcohol Alcohol.* Jul-Aug 1999;34(4):581-589.
8. Galvan FH, Caetano R. Alcohol use and related problems among ethnic minorities in the United States. *Alcohol research & health : the journal of the National Institute on Alcohol Abuse and Alcoholism.* 2003;27(1):87-94.
9. D'Onofrio G, Degutis LC. Integrating Project ASSERT: a screening, intervention, and referral to treatment program for unhealthy alcohol and drug use into an urban emergency department. *Acad Emerg Med.* Aug 2010;17(8):903-911.

10. Academic ED SBIRT Research Collaborative. The impact of screening, brief intervention and referral for treatment in emergency department patients' alcohol use: a 3-, 6- and 12-month follow-up. *Alcohol Alcohol.* Nov-Dec 2010;45(6):514-519.

11. Academic ED SBIRT Research Collaborative. The impact of screening, brief intervention, and referral for treatment on emergency department patients' alcohol use. *Ann Emerg Med.* Dec 2007;50(6):699-710, 710 e691-696.

12. Neumann T, Neuner B, Weiss-Gerlach E, Tonnesen H, Gentilello LM, Wernecke KD, Schmidt K, Schroder T, Wauer H, Heinz A, Mann K, Muller JM, Haas N, Kox WJ, Spies CD. The effect of computerized tailored brief advice on at-risk drinking in subcritically injured trauma patients. *J Trauma.* Oct 2006;61(4):805-814.

13. Longabaugh R, Woolard RE, Nirenberg TD, Minugh AP, Becker B, Clifford PR, Carty K, Licsw, Sparadeo F, Gogineni A. Evaluating the effects of a brief motivational intervention for injured drinkers in the emergency department. *J Stud Alcohol.* Nov 2001;62(6):806-816.

14. Havard A, Shakeshaft A, Sanson-Fisher R. Systematic review and meta-analyses of strategies targeting alcohol problems in emergency departments: interventions reduce alcohol-related injuries. *Addiction.* Mar 2008;103(3):368-376; discussion 377-368.

15. D'Onofrio G, Fiellin DA, Pantalon MV, Chawarski MC, Owens PH, Degutis LC, Busch SH, Bernstein SL, O'Connor PG. A Brief Intervention Reduces Hazardous and Harmful Drinking in Emergency Department Patients. *Ann Emerg Med.* Mar 28 2012.

16. Field CA, Caetano R, Harris TR, Frankowski R, Roudsari B. Ethnic differences in drinking outcomes following a brief alcohol intervention in the trauma care setting. *Addiction.* Jan 2010;105(1):62-73.

17. Desy PM, Perhats C. Alcohol screening, brief intervention, and referral in the emergency department: an implementation study. *J Emerg Nurs.* Feb 2008;34(1):11-19.

18. Monti PM, Barnett NP, Colby SM, Gwaltney CJ, Spirito A, Rohsenow DJ, Woolard R. Motivational interviewing versus feedback only in emergency care for young adult problem drinking. *Addiction.* Aug 2007;102(8):1234-1243.

19. Cunningham RM, Harrison SR, McKay MP, Mello MJ, Sochor M, Shandro JR, Walton MA, D'Onofrio G. National survey of emergency department alcohol screening and intervention practices. *Ann Emerg Med.* Jun 2010;55(6):556-562.

20. Cunningham RM, Walton MA, Goldstein A, Chermack ST, Shope JT, Bingham CR, Zimmerman MA, Blow FC. Three-month follow-up of brief computerized and therapist interventions for alcohol and violence among teens. *Acad Emerg Med.* Nov 2009;16(11):1193-1207.

21. Cunningham RM, Bernstein SL, Walton M, Broderick K, Vaca FE, Woolard R, Bernstein E, Blow F, D'Onofrio G. Alcohol, Tobacco, and Other Drugs: Future Directions for Screening and Intervention in the Emergency Department. *Academic Emergency Medicine.* Nov 2009;16(11):1078-1088.

22. Blow FC, Barry KL, Walton MA, Maio RF, Chermack ST, Bingham CR, Ignacio RV, Strecher VJ. The efficacy of two brief intervention strategies among injured,

at-risk drinkers in the emergency department: impact of tailored messaging and brief advice. *J Stud Alcohol.* Jul 2006;67(4):568-578.
23. Vaca FE, Winn D, Anderson C, Kim D, Arcila M. Feasibility of emergency department bilingual computerized alcohol screening, brief intervention, and referral to treatment. *Subst Abus.* Oct 2010;31(4):264-269.
24. Trinks A, Festin K, Bendtsen P, Nilsen P. Reach and effectiveness of a computer-based alcohol intervention in a Swedish emergency room. *International emergency nursing.* Jul 2010;18(3):138-146.
25. Nilsen P, Festin K, Guldbrandsson K, Carlfjord S, Holmqvist M, Bendtsen P. Implementation of a computerized alcohol advice concept in routine emergency care. *International emergency nursing.* Apr 2009;17(2):113-121.
26. Vaca FE, Winn D, Anderson C, Kim D, Arcila M. 6 month follow-up of computerized alcohol screening, brief intervention and referral to treatment in the emergency department. *Subst Abus.* 2011;32(3):144-152.
27. National Institute on Alcohol Abuse and Alcoholism. NIAAA's strategic plan to address health disparities. [Electronic]. http://pubs.niaaa.nih.gov/publications/HealthDisparities/Strategic.html. Accessed April 22, 2011.
28. Collins FS. Research agenda. Opportunities for research and NIH. *Science.* Jan 1 2010;327(5961):36-37.
29. Substance Abuse and Mental Health Services Administration. *Results from the 2009 National Survey on Drug Use and Health: Volume I. Summary of National Findings* Rockville, MD2010. HHS Publication No. SMA 10-4586 Findings.
30. U.S. Department of Health and Human Services. *10th Special Report to the U.S. Congress on Alcohol and Health.* Washington, DC: Public Health Service, National Institutes of Health, National Institute on Alcohol Abuse and Alcoholism;2000.
31. Mokdad AH, Marks JS, Stroup DF, Gerberding JL. Actual causes of death in the United States, 2000. *Jama.* Mar 10 2004;291(10):1238-1245.
32. Alcohol and suicide among racial/ethnic populations - 17 states, 2005-2006. *MMWR: Morbidity & Mortality Weekly Report.* 2009;58(23):637-641.
33. Center for Disease Control and Prevention. Alcohol-related disease impact (ARDI). 2008; http://www.cdc.gov/alcohol/ardi.htm.
34. Bouchery EE, Harwood HJ, Sacks JJ, Simon CJ, Brewer RD. Economic costs of excessive alcohol consumption in the U.S., 2006. *American journal of preventive medicine.* Nov 2011;41(5):516-524.
35. The George Washington University Medical Center, Ensuring Solutions to Alcohol Problems. Primer 1: Treating alcoholism as a chronic disease. n.d. www.ensuringsolutions.org/usr_doc/PDF_Version_of_Primer.pdf. Accessed April 13, 2011.
36. Mangione TW, Howland J, Lee M. *New perspectives for worksite alcohol strategies: Results from a corporate drinking study. .* Boston, MA: JSI Research and Triangle Institute.; 1998.
37. Lowenstein SR, Weissberg MP, Terry D. Alcohol intoxication, injuries, and dangerous behaviors--and the revolving emergency department door. *J Trauma.* Oct 1990;30(10):1252-1258.

38. Alcohol-attributable deaths and years of potential life lost -- United States, 2001. *MMWR: Morbidity & Mortality Weekly Report.* 2004;53(37):866.

39. Freedland ES, McMicken DB, D'Onofrio G. Alcohol and trauma. *Emerg Med Clin North Am.* Feb 1993;11(1):225-239.

40. Lieber CS. Medical disorders of alcoholism. *N Engl J Med.* Oct 19 1995;333(16):1058-1065.

41. Rosen SM, Miller TR, Simon M. The cost of alcohol in California. *Alcoholism, clinical and experimental research.* Nov 2008;32(11):1925-1936.

42. Pew Hispanic Center. *Census 2010, 50 million Latinos: Hispanics account for more than half of nation's growth in past decade* Washington, DC: Pew Research Center; March 24 2011.

43. Humes KR, Jones NA, Ramirez RR. *Overview of Race and Hispanic Origin: 2010, 2010 Census Brief.* Washington, D.C: U.S. Census Bureau;2011.

44. US Census Bureau, US Department of Commerce. *US Census Bureau News: Profile America, Facts for Features.* Washington DC March 12 2012. CB12-FF.10.

45. U.S. Department of Health and Human Services, Office of Disease Prevention and Health Promotion. Healthy People 2020. http://www.healthypeople.gov/2020/Consortium/HP2020Framework.pdf. Accessed April 10, 2012.

46. National Institute on Alcohol Abuse and Alcoholism. *Alcohol and the Hispanic Community.* Bethesda, MD: National Institutes of Health; April 2011 2011.

47. Chartier KG, Caetano R. Trends in alcohol services utilization from 1991-1992 to 2001-2002: ethnic group differences in the U.S. population. *Alcoholism, clinical and experimental research.* Aug 2011;35(8):1485-1497.

48. Caetano R, Clark CL, Tam T. Alcohol consumption among racial/ethnic minorities: theory and research. *Alcohol health and research world.* 1998;22(4):233-241.

49. Caetano R, Ramisetty-Mikler S, Rodriguez LA. The Hispanic Americans Baseline Alcohol Survey (HABLAS): Rates and predictors of DUI across Hispanic national groups. *Accid Anal Prev.* Mar 2008;40(2):733-741.

50. Campos-Outcalt D, Bay C, Dellapenna A, Cota MK. Pedestrian fatalities by race/ethnicity in Arizona, 1990-1996. *American journal of preventive medicine.* Aug 2002;23(2):129-135.

51. Grant BF, Hasin DS, Stinson FS, Dawson DA, Chou SP, Ruan WJ, Pickering RP. Prevalence, correlates, and disability of personality disorders in the United States: results from the national epidemiologic survey on alcohol and related conditions. *The Journal of clinical psychiatry.* Jul 2004;65(7):948-958.

52. Alcohol Research Group and National Institute on Alcohol Abuse and Alcoholism (NIAAA). *National Alcohol Survey: 1984.* Bethesda, MD: U.S. Department of Health and Human Services

53. Caetano R, Clark CL. Trends in alcohol consumption patterns among whites, blacks and Hispanics: 1984 and 1995. *J Stud Alcohol.* Nov 1998;59(6):659-668.

54. Caetano R, Clark CL. Trends in alcohol-related problems among whites, blacks, and Hispanics: 1984-1995. *Alcoholism, clinical and experimental research.* Apr 1998;22(2):534-538.

55. National Institute on Alcohol Abuse and Alcoholism. *Alcohol use and alcohol use disorders in the United States: Main findings from the 2001-2002 National Epidemiologic Survey on Alcohol and Related Conditions (NESARC).* Bethesda, MD: National Institutes of Health;2006.

56. Caetano R. Prevalence, incidence and stability of drinking problems among whites, blacks and Hispanics: 1984-1992. *J Stud Alcohol.* Nov 1997;58(6):565-572.

57. Substance Abuse and Mental Health Services Administration. *Results from the 2010 NationalSurvey on Drug Use and Health: Summary of National Findings, NSDUH Series H-41.* Rockville, MD: Substance Abuse and Mental Health Services Administration;2011. HHS Publication No. (SMA) 11-4658.

58. Hasin DS, Stinson FS, Ogburn E, Grant BF. Prevalence, correlates, disability, and comorbidity of DSM-IV alcohol abuse and dependence in the United States: results from the National Epidemiologic Survey on Alcohol and Related Conditions. *Arch Gen Psychiatry.* Jul 2007;64(7):830-842.

59. Dawson DA, Grant BF, Stinson FS, Chou PS, Huang B, Ruan WJ. Recovery from DSM-IV alcohol dependence: United States, 2001-2002. *Addiction.* Mar 2005;100(3):281-292.

60. Caetano R. Alcohol-related health disparities and treatment-related epidemiological findings among whites, blacks, and Hispanics in the United States. *Alcoholism, clinical and experimental research.* Aug 2003;27(8):1337-1339.

61. Caetano R, Clark CL. Hispanics, Blacks and White driving under the influence of alcohol: results from the 1995 National Alcohol Survey. *Accid Anal Prev.* Jan 2000;32(1):57-64.

62. Caetano R, Mills BA. The Hispanic Americans Baseline Alcohol Survey (HABLAS): Is the "Prevention Paradox" Applicable to Alcohol Problems Across Hispanic National Groups? *Alcoholism, clinical and experimental research.* Mar 15 2011.

63. Mills BA, Caetano R. The Hispanic Americans Baseline Alcohol Survey (HABLAS): predictors of alcohol attitudes and expectancies in Hispanic national groups. *Alcoholism, clinical and experimental research.* May 2010;34(5):790-799.

64. Vaeth PA, Caetano R, Ramisetty-Mikler S, Rodriguez LA. Hispanic Americans Baseline Alcohol Survey (HABLAS): alcohol-related problems across Hispanic national groups. *J Stud Alcohol Drugs.* Nov 2009;70(6):991-999.

65. Caetano R, Vaeth PA, Rodriguez LA. The Hispanic Americans Baseline Alcohol Survey (HABLAS): Acculturation, Birthplace and Alcohol-Related Social Problems Across Hispanic National Groups. *Hispanic journal of behavioral sciences.* Feb 1 2012;31(1):95-117.

66. US Census Bureau, US Department of Commerce. *US Census Bureau News: Profile America, Facts for Features: Hispanic Heritage Month 2011 - Sept. 15 - Oct 15.* Washington DC August 26, 2011 2011. CB11-FF.18.

67. National Hospital Ambulatory Medical Care Survey 2008 Emergency Department Summary Tables 2010. http://www.cdc.gov/nchs/ahcd/web_tables.htm. Accessed May 15, 2012.

68. Niska R, Bhuiya F, Xu J. *National Hospital Ambulatory Medical Care Survey 2007 Emergency Department Summary.* Washington, D.C.: U.S. Department of Health and Human Services, Centers for Disease Control and Prevention, National Center for Health Statistics;2010.
69. National Hospital Ambulatory Medical Care Survey 2009 Emergency Department Summary Tables 2011. http://www.cdc.gov/nchs/ahcd/web_tables.htm. Accessed May 15, 2012.
70. Cherpitel CJ. Breath analysis and self-reports as measures of alcohol-related emergency room admissions. *J Stud Alcohol.* Mar 1989;50(2):155-161.
71. McDonald AJ, 3rd, Wang N, Camargo CA, Jr. US emergency department visits for alcohol-related diseases and injuries between 1992 and 2000. *Arch Intern Med.* Mar 8 2004;164(5):531-537.
72. Bernstein E, Tracey A, Bernstein J, Williams C. Emergency Department Detection and Referral Rates for Patients with Problem Drinking. *Substance Abuse.* 1996;17(2):69 - 76.
73. Current Research in Alcohol. *Annals of Emergency Medicine.* 1997;30(6):817-819.
74. D'Onofrio G, Becker B, Woolard RH. The impact of alcohol, tobacco, and other drug use and abuse in the emergency department. *Emerg Med Clin North Am.* Nov 2006;24(4):925-967.
75. Degutis LC. Screening for Alcohol Problems in Emergency Department Patients with Minor Injury: Results and Recommendations for Practice and Policy. *Contemp Drug Probs.* 1998;25:463-475.
76. Saunders JB, Aasland OG, Babor TF, de la Fuente JR, Grant M. Development of the Alcohol Use Disorders Identification Test (AUDIT): WHO Collaborative Project on Early Detection of Persons with Harmful Alcohol Consumption--II. *Addiction.* Jun 1993;88(6):791-804.
77. Willenbring ML, Massey SH, Gardner MB. Helping patients who drink too much: an evidence-based guide for primary care clinicians. *Am Fam Physician.* Jul 1 2009;80(1):44-50.
78. National Institute on Alcohol Abuse and Alcoholism. *Helping Patients Who Drink Too Much : A Clinician's Guide Updated 2005 Edition* US Department of Health and Human Services, National Institutes of Health 2007 (Reprinted). NIH Publication No. 07-3769.
79. Institute of Medicine. *Broadening the Base of Treatment for Alcohol Problems: Report of a Study by a Committee of the Institute of Medicine.* Washington, DC: Division of Mental Health and Behavioral Medicine;1990.
80. Cherpitel CJ. Screening for Alcohol Problems in the Emergency Department. *Annals of Emergency Medicine.* 1995;26(2):158-166.
81. Cherpitel CJ. Drinking Patterns and Problems: A Comparison of Primary Care with the Emergency Room. *Subst Abus.* Jun 1999;20(2):85-95.
82. Davidson P, Koziol-McLain J, Harrison L, Timken D, Lowenstein SR. Intoxicated ED patients: a 5-year follow-up of morbidity and mortality. *Ann Emerg Med.* Nov 1997;30(5):593-597.
83. Mertens JR, Lu YW, Parthasarathy S, Moore C, Weisner CM. Medical and psychiatric conditions of alcohol and drug treatment patients in an HMO:

comparison with matched controls. *Arch Intern Med.* Nov 10 2003;163(20):2511-2517.
84. Kraemer KL. The cost-effectiveness and cost-benefit of screening and brief intervention for unhealthy alcohol use in medical settings. *Subst Abus.* 2007;28(3):67-77.
85. Solberg LI, Maciosek MV, Edwards NM. Primary care intervention to reduce alcohol misuse ranking its health impact and cost effectiveness. *American journal of preventive medicine.* Feb 2008;34(2):143-152.
86. Fleming MF, Mundt MP, French MT, Manwell LB, Stauffacher EA, Barry KL. Benefit-cost analysis of brief physician advice with problem drinkers in primary care settings. *Med Care.* Jan 2000;38(1):7-18.
87. Gentilello LM, Ebel BE, Wickizer TM, Salkever DS, Rivara FP. Alcohol interventions for trauma patients treated in emergency departments and hospitals: a cost benefit analysis. *Ann Surg.* Apr 2005;241(4):541-550.
88. Spirito A, Monti PM, Barnett NP, Colby SM, Sindelar H, Rohsenow DJ, Lewander W, Myers M. A randomized clinical trial of a brief motivational intervention for alcohol-positive adolescents treated in an emergency department. *The Journal of Pediatrics.* 2004/9 2004;145(3):396-402.
89. Monti PM, Colby SM, Barnett NP, Spirito A, Rohsenow DJ, Myers M, Woolard R, Lewander W. Brief intervention for harm reduction with alcohol-positive older adolescents in a hospital emergency department. *J Consult Clin Psychol.* Dec 1999;67(6):989-994.
90. Crawford MJ, Patton R, Touquet R, Drummond C, Byford S, Barrett B, Reece B, Brown A, Henry JA. Screening and referral for brief intervention of alcohol-misusing patients in an emergency department: a pragmatic randomised controlled trial. *The Lancet.* 2004;364(9442):1334-1339.
91. Becker B, Woolard R, Nirenberg TD, Minugh PA, Longabaugh R, Clifford PR. Alcohol Use among Subcritically Injured Emergency Department Patients. *Academic Emergency Medicine.* 1995;2(9):784-790.
92. Study P, Cloud RN, Peacock PL. Internet Screening and Interventions for Problem Drinking: Results from the http://www.carebetter.com. *Alcoholism Treatment Quarterly.* 2001;19(2):23 - 44.
93. Reinert DF, Allen JP. The Alcohol Use Disorders Identification Test (AUDIT): a review of recent research. *Alcoholism, clinical and experimental research.* Feb 2002;26(2):272-279.
94. Chan-Pensley E. Alcohol-Use Disorders Identification Test: a comparison between paper and pencil and computerized versions. *Alcohol Alcohol.* Nov-Dec 1999;34(6):882-885.
95. Walters ST, Wright JA, Shegog R. A review of computer and Internet-based interventions for smoking behavior. *Addict Behav.* Feb 2006;31(2):264-277.
96. Hester RK, Delaney HD, Campbell W. The college drinker's check-up: outcomes of two randomized clinical trials of a computer-delivered intervention. *Psychology of addictive behaviors : journal of the Society of Psychologists in Addictive Behaviors.* Mar 2012;26(1):1-12.

97. Schinke SP, Schwinn TM, Ozanian AJ. Alcohol abuse prevention among high-risk youth: computer-based intervention. *J Prev Interv Community.* 2005;29(1-2):117-130.

98. Carey KB, Scott-Sheldon LA, Elliott JC, Bolles JR, Carey MP. Computer-delivered interventions to reduce college student drinking: a meta-analysis. *Addiction.* Nov 2009;104(11):1807-1819.

99. Carey KB, Henson JM, Carey MP, Maisto SA. Computer versus in-person intervention for students violating campus alcohol policy. *J Consult Clin Psychol.* Feb 2009;77(1):74-87.

100. Butler S, Chiauzzi E, Bromberg JI, Budman SH, Buono DP. Computer assisted screening and intervenion for alcohol problems in primary care. *Journal of Technology and Human Services.* 2003;21(3):1-19.

101. Dimeff LA, McNeely M. Computer-enhanced primary care practitioner advice for high-risk college drinkers in a student primary health-care setting. *Cognitive and Behavioral Practice.* 2000;7(1):82-100.

102. Kypri K, Langley JD, Saunders JB, Cashell-Smith ML, Herbison P. Randomized controlled trial of web-based alcohol screening and brief intervention in primary care. *Arch Intern Med.* Mar 10 2008;168(5):530-536.

103. Gregor MA, Shope JT, Blow FC, Maio RF, Weber JE, Nypaver MM. Feasibility of using an interactive laptop program in the emergency department to prevent alcohol misuse among adolescents. *Ann Emerg Med.* Aug 2003;42(2):276-284.

104. Karlsson A, Bendtsen P. Acceptability of a computerized alcohol screening and advice routine in an emergency department setting--a patient perspective. *Addict Behav.* May 2005;30(4):767-776.

105. Hungerford DW, Pollock DA editors. *Alcohol Problems Among Emergency Department Patients: Proceedings of a Research Conference on Identification and Intervention.* Atlanta, GA: National Center for Injury Prevention and Control, Centers for Disease Control and Prevention;2002.

106. Cherpitel CJ. Screening and brief intervention for alcohol problems in the emergency room: is there a role for nursing? *Journal of Addictions Nursing.* 2006;17(2):79-82.

107. Vaca FE, Winn D. The basics of alcohol screening, brief intervention and referral to treatment in the emergency department. *West J Emerg Med.* Aug 2007;8(3):88-92.

108. Cherpitel CJ. Analysis of cut points for screening instruments for alcohol problems in the emergency room. *J Stud Alcohol.* Nov 1995;56(6):695-700.

109. Cherpitel CJ. Comparison of screening instruments for alcohol problems between black and white emergency room patients from two regions of the country. *Alcoholism, clinical and experimental research.* Nov 1997;21(8):1391-1397.

110. De Leeuw E, Hox J, Kef S. Computer-Assisted Self-Interviewing Tailored for Special Populations and Topics. *Field Methods.* Aug 2003;15(3):223-251.

111. Marin G, Marin B. Hispanics: Who are they? *Research with Hispanic Populations.* Vol 23. Newbury Park, California: SAGE Publications, Inc. ; 1991:12-13.

112. Tourangeau R, Smith TW. Asking Sensitive Questions: The Impact of Data Collection Mode, Question Format, and Question Context. *The Public Opinion Quarterly.* 1996;60(2):275-304.

113. D'Onofrio G, Bernstein E, Rollnick S. Motivating patients for change: a brief strategy for negotiation. In: Bernstein E, Bernstein J, eds. *Case studies in Emergency Medicine and the health of the Public* Boston: Jones and Bartlett; 1996:295-303.

114. Prochaska JO, DiClemente CC, Norcross JC. In search of how people change: Applications of addictive behaviors. *Am Psychologist.* 1992;47(9):1102-1114.

115. U.S. Department of Health and Human Services. Healthy people 2020 topics and objectives: Substance abuse. 2011; http://www.healthypeople.gov/2020/topicsobjectives2020/objectiveslist.aspx?topicid=40. Accessed April 24, 2011.

116. D'Onofrio G, Pantalon MV, Degutis LC, Fiellin DA, Busch SH, Chawarski MC, Owens PH, O'Connor PG. Brief intervention for hazardous and harmful drinkers in the emergency department. *Ann Emerg Med.* Jun 2008;51(6):742-750 e742.

117. Babor TF, Higgins-Biddle JC, Saunders JB, Monteiro MG. *The Alcohol Use Disorders Identification Test: Guidelines for Use in Primary Care.* Geneva, Switzerland: World Health Organization, Department of Mental Health and Substance Dependence;2001.

118. Maio RF, Shope JT, Blow FC, Gregor MA, Zakrajsek JS, Weber JE, Nypaver MM. A randomized controlled trial of an emergency department-based interactive computer program to prevent alcohol misuse among injured adolescents. *Ann Emerg Med.* Apr 2005;45(4):420-429.

119. Daeppen JB, Gaume J, Bady P, Yersin B, Calmes JM, Givel JC, Gmel G. Brief alcohol intervention and alcohol assessment do not influence alcohol use in injured patients treated in the emergency department: a randomized controlled clinical trial. *Addiction.* Aug 2007;102(8):1224-1233.

120. Caetano R, Mills BA, Harris TR. Hispanic Americans Baseline Alcohol Survey (HABLAS): effects of container size adjustments on estimates of alcohol consumption across Hispanic national groups. *J Stud Alcohol Drugs.* Jan 2012;73(1):120-125.

121. Fleming MF, Bruno M, Barry K, Fost N. Informed consent, deception, and the use of disguised alcohol questionnaires. *Am J Drug Alcohol Abuse.* 1989;15(3):309-319.

122. Fleming MF, Barry KL. A three-sample test of a masked alcohol screening questionnaire. *Alcohol Alcohol.* 1991;26(1):81-91.

123. D'Onofrio G, Fiellin DA, Pantalon MV, Degutis LC, Owens P, Bernstein S, Busch SH, Chawarski MC, O'Connor P. Brief interventions for hazardous and harmful drinkers in the ED. *Acad Emerg Med* 2010;S75.

124. D'Onofrio G, Fiellin DA, Pantalon MV, Degutis LC, Owens P, Bernstein S, Busch SH, Chawarski MC, O'Connor P. Emergency physician performed interventions to reduce hazardous and harmful alcohol use in the emergency department. *Unpublished (NIAAA #1R01AA14963: Trial Registration clinicaltrials.gov Identifier: NCT00261859).* 2011.

125. Polsky D, Glick H. Non-Study Medical Services Form (NSMS). Personal Communication,2004.

126. Starfield B. Injury behavior checklist (adapted version) Subscale, Adolescent Health Status Instrument Baltimore, MD: John Hopkins University; 1991.

127. Miller WR, Tonigan JS, Longabaugh R. *The Drinker Inventory of Consequences (DrInC): An instrument for assessing adverse consequences of alcohol abuse. Test manual (Project MATCH Monograph Series Vol 4).* Rockville, MD National Institute on Alcohol Abuse and Alcoholism;1995.

128. Cohen BB, Vinson DC. Retrospective self-report of alcohol consumption: test-retest reliability by telephone. *Alcoholism, clinical and experimental research.* Oct 1995;19(5):1156-1161.

129. Breslin C, Sobell LC, Sobell MB, Buchan G, Kwan E. Aftercare telephone contacts with problem drinkers can serve a clinical and research function. *Addiction.* Sep 1996;91(9):1359-1364.

130. McLellan TA, Zanis D, Incmikoski R. *Treatment Service Review (TSR).* Philadelphia: The Center for Studies in Addiction, Department of Psychiatry: Philadelphia VA Medical Center & The University of Pennsylvania; 1989.

131. American College of Surgeons - Committee on Trauma. Alcohol Screening and Brief Intervention (SBI) Training for Trauma Patients: COT Quick Guide. http://www.facs.org/trauma/publications/sbirtguide.pdf. Accessed May 15, 2011.

132. Fleming MF, Barry KL, Manwell LB, Johnson K, London R. Brief physician advice for problem alcohol drinkers. A randomized controlled trial in community-based primary care practices. *JAMA.* Apr 2 1997;277(13):1039-1045.

133. O'Malley SS, Jaffe AJ, Chang G, Rode S, Schottenfeld R, Meyer RE, Rounsaville B. Six-month follow-up of naltrexone and psychotherapy for alcohol dependence. *Arch Gen Psychiatry.* Mar 1996;53(3):217-224.

134. Carroll KM, Rounsaville BJ, Nich C, Gordon LT, Wirtz PW, Gawin F. One-year follow-up of psychotherapy and pharmacotherapy for cocaine dependence. Delayed emergence of psychotherapy effects. *Arch Gen Psychiatry.* Dec 1994;51(12):989-997.

135. Woolard RH, Carty K, Wirtz P, Longabaugh R, Nirenberg TD, Minugh PA, Becker B, Clifford PR. Research fundamentals: follow-up of subjects in clinical trials: addressing subject attrition. *Acad Emerg Med.* Aug 2004;11(8):859-866.

136. Ribisl KM, Walton MA, Mowbray CT, Luke DA, Davidson Ii WS, Bootsmiller BJ. Minimizing participant attrition in panel studies through the use of effective retention and tracking strategies: Review and recommendations. *Evaluation and Program Planning.* 1996;19(1):1-25.

137. Whitlock EP, Polen MR, Green CA, Orleans T, Klein J. Behavioral Counseling Interventions in Primary Care To Reduce Risky/Harmful Alcohol Use by Adults: A Summary of the Evidence for the U.S. Preventive Services Task Force. *Ann Intern Med.* April 6, 2004 2004;140(7):557-568.

138. Kaner EF, Beyer F, Dickinson HO, Pienaar E, Campbell F, Schlesinger C, Heather N, Saunders J, Burnand B. Effectiveness of brief alcohol interventions in primary care populations. *Cochrane Database Syst Rev.* 2007(2):CD004148.

139. Dmitrienko A, Molenberghs G, Chuang-Stein C, Offen W. *Analysis of Clinical Trials using SAS: A Practical Guide.* Cary, NC: SAS Institute, Inc.; 2005.

140. Molenberghs G, Thijs H, Jansen I, Beunckens C, Kenward MG, Mallinckrodt C, Carroll RJ. Analyzing incomplete longitudinal clinical trial data. *Biostatistics.* Jul 2004;5(3):445-464.

141. Committee on National Statistics Division of Behavioral and Social Sciences and Education. *The Prevention and Treatment of Missing Data in Clinical Trials. Panel on Handling Missing Data in Clinical Trials.* Washington, DC National Research Council;2010.

142. Lachin JM. Statistical considerations in the intent-to-treat principle. *Control Clin Trials.* Jun 2000;21(3):167-189.

143. Schrimsher GW, Filtz K. Assessment Reactivity: Can Assessment of Alcohol Use During Research be an Active Treatment? *Alcoholism Treatment Quarterly.* 2011;29(2):108 - 115.

STATISTICAL ANALYSIS PLAN

AUTOMATED BILINGUAL COMPUTERIZED ALCOHOL SCREENING AND INTERVENTION IN LATINOS (AB-CASI)

VERSION 1.0
MARCH 26, 2020

**FUNDING AGENCIES:**	**NIAAA**
**NIA GRANT NUMBER:**	**R01AA022083-01A1**
**CLINICALTRIALS.GOV IDENTIFIER**	**NCT02247388**
**PRINCIPAL INVESTIGATOR**	**FEDERICO VACA, MD**

Prepared by:

**James Dziura, PhD**	**(blinded study biostatistician)**
**Jesse Reynolds, MS**	**(unblinded study biostatistician)**
**Kaitlin Maciejewski, MS**	**(unblinded study biostatistician)**

The SAP is a living document and will be modified as necessary up to final data lock and before study unblinding.

Table of Contents

1. Study Synopsis
2. Study Objectives and Specific Aims
3. Randomization
4. Blinding
5. Endpoints
6. Sample Size
7. Interim Monitoring
8. Data Collection and Data Freeze
9. Analytic Plan
References

1. STUDY SYNOPSIS

Title	**AUTOMATED BILINGUAL COMPUTERIZED ALCOHOL SCREENING AND INTERVENTION IN LATINOS (AB-CASI)**
Study Design	The design is a randomized 2-arm superiority trial. The unit of randomization is the patient.
Study Duration	6 years
Trial Sites	1 trial site: Bridgeport Hospital Emergency Department
Objective	Conduct a randomized controlled trial to determine the efficacy of AB-CASI compared to standard care (SC) in the reduction of alcohol consumption, negative behavior and 30-day treatment engagement in unhealthy drinkers.
Number of Subjects	840: 420 in AB-CASI, 420 in SC
Inclusion Criteria	• English and Spanish speaking adult<br>• ≥ 18 y/o<br>• Latino patients who present to the Bridgeport Hospital's ED<br>• Drink over the NIAAA low-risk limits.
Exclusion Criteria	• Primary language other than English or Spanish<br>• Current enrollment in an alcohol or substance abuse treatment program<br>• Current ED visit for acute psychosis;<br>• Condition that precludes interview or AB-CASI use i.e., life threatening injury/illness<br>• In police custody<br>• Inability to provide two contact numbers for follow-up.

Interventions	**<u>AB-CASI</u>:** AB-CASI is a bilingual (English and Spanish) automated interactive audiographical interface software program that was developed for automated ED-SBIRT. This program will run on an iPad® and will be provided to an ED patient that is initially screened and found to be drinking above the NIAAA low-risk limits. Prior to providing the iPad® to the patient, the RA will briefly introduce the purpose of AB-CASI to the patient. Thereafter, the iPad® will be given to the patient with its accompanying headphones and the program is started. AB-CASI directs the patient to listen to the brief self-contained program instructions. Once AB-CASI asks permission of the patient to <u>RAISE THE SUBJECT</u> to discuss alcohol use in their lives and the patient agrees, a series of alcohol and health-related questions and messages are displayed on the iPad® and spoken through the headphones in the language preference (English or Spanish). After AB-CASI interactively orients the patient to the definition of a "standard drink", they are asked about their alcohol consumption according to quantity and frequency and then taken to the embedded AUDIT portion of the program. Through logic branching and automated tabulation of responses to the AUDIT, the patient is <u>PROVIDED FEEDBACK</u> via an AUDIT score with its respective definition along with the NIAAA guidelines for low-risk for reference. The patient will additionally be informed of how their consumption level compares to that of other Latino drinkers throughout the U.S. Thereafter, the patient receives the remaining components of the AB-CASI BNI that encompasses <u>ENHANCING MOTIVATION</u> by discussing their readiness to change their drinking pattern to one that is safer and their reasons for cutting down. The motivation enhancement for the dependent drinker is centered on seeking specialized treatment services. Finally, <u>NEGOTIATING AND ADVICE</u> is provided interactively by AB-CASI and the patient is encouraged to set a goal for reducing their alcohol consumption and a personalized alcohol reduction plan is printed and provided to the patient at the conclusion of the AB-CASI encounter. The dependent drinker will receive a personalized plan focused on referral to treatment and encouraged to intentionally engage in specialized treatment at a local facility. Our previous work has shown that the AB-CASI program encounter is highly acceptable to ED adult patients and takes an average of ≤ 10 minutes. Further, our extensive experience with AB-CASI has shown it to be user friendly to patients with the most basic computer skills. While our experience with AB-CASI has shown us that patients do not usually need any significant level of help with navigating AB-CASI, the RA will be available for any questions the ED patient might have regarding its use.<br><br>**<u>Standard Care (SC)</u>:** Patients randomized to SC will not receive AB-CASI. However, they will receive SC as provided by the treating emergency medicine physician at Bridgeport Hospital. All SC patients will receive an informational sheet with primary care follow-up recommended. All requirements for <u>screening and referral</u> will be performed according to the American College of Surgeons (ACS) Level 2 trauma designation. The mandate for <u>brief intervention</u> by the

	ACS applies only to Level 1 trauma centers and not to Level 2 trauma centers like Bridgeport Hospital. In general, quantitative assessments of alcohol levels are completed only on patients designated as a full-trauma response which is less than 3% of all the visits for the entire ED (>77,000/yr.). Consultation with social workers are at the discretion of the treating physician. Essentially all the physicians working in the Bridgeport Hospital ED are not Spanish speaking and have minimal to no training in alcohol SBIRT. Further, given the reflective nature of questioning required for the BNI, ED physicians that have some working knowledge of "medical-Spanish", lack the proficiency to effectively perform alcohol screening and BNI. In our study, patients will be briefly screened by RAs as part of an overall health screen, and the treating ED physician will have no knowledge of these results. In order to assess the nature of the care provided by the ED physician, we will review the ED record of each enrolled study patient assigned to standard condition and code for physician-initiated assessment, any intervention and/or referral to treatment (i.e. any documented discussion about alcohol use or referral to treatment facility in the ED treatment record or discharge instructions). Finally, while the Yale research team has had a long-standing collaborative relationship with the Bridgeport Hospital Emergency Department, no observational or randomized clinical trials of ED-SBIRT have been previously undertaken at this site. Therefore, we believe that the risk of contamination to the standard care as a result of previous research efforts is non-existent.
Duration of Intervention and Follow-up	12 months
Primary Outcome	Self-reported number of binge drinking episodes over the previous 28 days assessed using the 28-day timeline follow back (assessed 12 months after randomization)
Primary Analysis	All analyses will be according to intent to treat. A generalized linear mixed model (GLMM) with a Negative Binomial distribution will be used to estimate differences in the number of binge drinking episodes in the past 28 days. The mixed model will include fixed effects for intervention (AB-CASI vs. SC), time (1, 6, 12 months), and the interaction of intervention with time. Additional fixed effects will be included for baseline covariates (baseline number of drinks per week, baseline number of binge episodes, gender, English vs. Spanish preferred language and dependence status).

Secondary Outcomes	• Self reported mean number of weekly drinks as measured by a 28 day timeline follow back (assessed 12 months after randomization)<br>• Episodes of Impaired Driving over 12 months [Alcohol-Related Negative Health Behaviors and Consequences]<br>• Episodes of Riding with an Impaired Driver over 12 months [Alcohol-Related Negative Health Behaviors and Consequences]<br>• Injuries over 12 months [Alcohol-Related Negative Health Behaviors and Consequences]<br>• Arrests over 12 months [Alcohol-Related Negative Health Behaviors and Consequences]<br>• Tardiness over 12 months [Alcohol-Related Negative Health Behaviors and Consequences]<br>• Days Absent from Work or School over 12 months [Alcohol-Related Negative Health Behaviors and Consequences]
Tertiary Outcomes	30 Day Treatment Engagement
Interim Analysis	No monitoring for efficacy or futility is proposed; only monitoring for safety and study conduct will be done.

2. STUDY OBJECTIVES AND SPECIFIC AIMS

AB-CASI is a randomized controlled trial to determine the efficacy of AB-CASI compared to standard care (SC) in the reduction of alcohol consumption, negative health behaviors and consequences, and 30-day treatment engagement in unhealthy drinkers.

The study's Specific Aims and Hypotheses are:

Aim 1. To compare the efficacy of AB-CASI (Automated Bilingual Computerized Alcohol Screening and Intervention) to SC (standard care) in the reduction of alcohol consumption in unhealthy drinkers.

Hypothesis 1. At 12 months, AB-CASI will be superior to SC in reducing the number of binge drinking episodes and the mean number of weekly drinks over the last 28-days.

Aim 2. To compare the efficacy of AB-CASI to SC in the reduction of alcohol-related negative health behaviors and consequences.

Hypothesis 2. At 12 months, AB-CASI will be superior to SC in reducing alcohol-related negative health behaviors and consequences (episodes of impaired driving, riding with an impaired driver, injuries, arrests, tardiness and days absent from work/school).

Aim 3. To compare the efficacy of AB-CASI to SC in 30-day treatment engagement.

Hypothesis 3. AB-CASI will be superior to SC in increasing 30-day treatment engagement in unhealthy drinkers.

Exploratory Aim. To explore variation of AB-CASI on alcohol consumption, alcohol-related negative health behaviors and consequences and 30-day treatment engagement across Latino subpopulations (Puerto-Rican, Mexican-American, Cuban-American, South/Central American) as well as other potential modifiers (age, birthplace, gender, preferred language, dependence, reason for ED visit, and smoking status).

3. RANDOMIZATION

Unhealthy drinkers of all severity that fulfill all enrollment criteria and consent to study participation will have their baseline assessments performed and will be immediately randomized to one of the two groups (AB-CASI or SC) in a 1:1 ratio. ***Participants will be randomized using a stratified randomization procedure [stratification by dependence status (AUDIT<20 vs. AUDIT≥20) AND by English vs. Spanish AB-CASI patient preference].*** Random permuted blocks (size 4 and 6) will be used to assure equal allocation. The randomization scheme is computer generated, concealed and delivered by Oncore (the clinical trials data management system).

4. BLINDING

AB-CASI is an unblinded trial. However, all baseline and follow-up data collection will be completed by blinded site staff.

At the Data Coordinating Center, Dr. Dziura will be the blinded statistician who will interface with the study leadership and attend the open session of the DSMB meetings; Mr. Jesse Reynolds and Ms. Kaitlin Maciejewski will be the unblinded statisticians who will prepare the randomization scheme and the closed DSMB report as well as attend the closed DSMB sessions.

5. OUTCOME MEASURES

5.1 Schedule of Assessments

The assessment schedule is shown in the figure below.

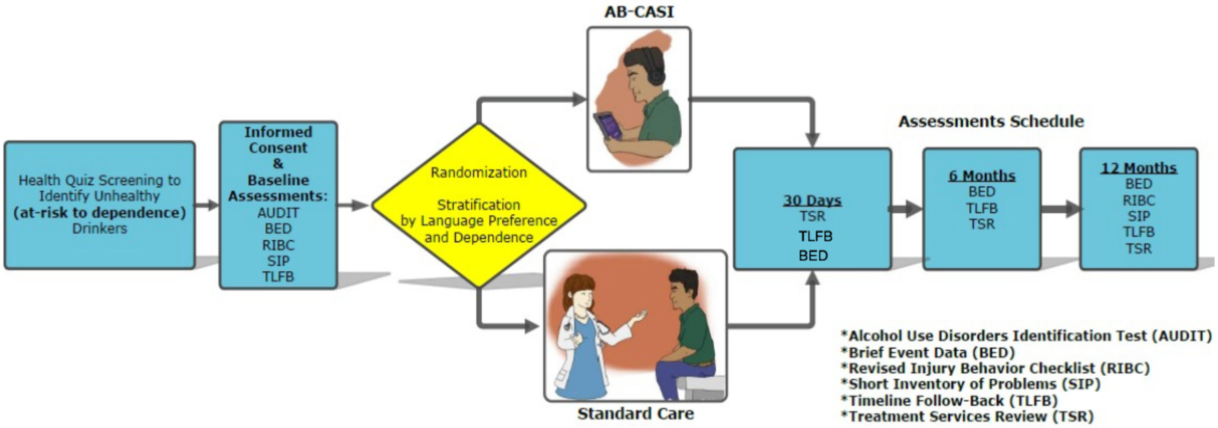

5.2 Primary Outcomes

1. Self-Reported Binge Drinking Episodes

The primary outcome is Self-Reported Binge Drinking Episodes at 12 months post-randomization. The outcome is assessed using the 28-day Timeline Follow Back (TLFB) at 1 month, 6 months and 12 months.

5.3 Secondary Outcomes

1. Mean Number of Weekly Drinks

Self-reported mean number of weekly drinks at 12 months post-randomization is assessed using the 28-day TLFB. This outcome is assessed at 1 month, 6 months and 12 months.

Secondary Outcomes 2-7 below are assessed via the Brief Event Data survey at 1 month, 6 and 12 months.

2. Episodes of Impaired Driving over 12 months [Alcohol-Related Negative Health Behaviors and Consequences]

3. Episodes of Riding with an Impaired Driver over 12 months [Alcohol-Related Negative Health Behaviors and Consequences]

4. Injuries over 12 months [Alcohol-Related Negative Health Behaviors and Consequences]

5. Arrests over 12 months [Alcohol-Related Negative Health Behaviors and Consequences]

6. Tardiness over 12 months [Alcohol-Related Negative Health Behaviors and Consequences]

7. Days Absent from Work or School over 12 months [Alcohol-Related Negative Health Behaviors and Consequences]

5.5 Exploratory Outcomes

1. 30-day treatment engagement

This outcome will be assessed by subject self-report and verified by the RA through contact with the specified treatment program. Patients will be considered to be engaged in treatment if at 30-days after randomization the patient reports receiving care in a treatment program that addresses their AUD (e.g. outpatient or inpatient detoxification, therapeutic community). Participation in a self-help program (e.g. A.A.) will also be considered as treatment engagement.

2. 30-day treatment contact

This outcome will be assessed by subject self-report and verified by the RA through contact with the specified treatment program. Patients will be considered to have contacted a treatment facility or program if at 30-days after randomization the patient reports contacting or a treatment program that addresses their AUD (e.g. outpatient or inpatient detoxification, therapeutic community). Participation in treatment program (including self-help) will also be considered as treatment contact.

6. SAMPLE SIZE

6.1 Primary Outcome

Estimation of sample size is based on randomizing and following a sufficient number of unhealthy drinkers to evaluate the primary hypothesis (Aim 1). The primary hypothesis is that AB-CASI will result in greater 12-month reductions in the primary outcome, the number of episodes of binge drinking over the past 28-days, which is assessed using the 28-day timeline follow-back method, when compared to SC. Fleming et al.[132] demonstrated that the number of binge episodes in the past 30-days was reduced by 1.14 in the intervention compared to control conditions. D'Onofrio et al.[15] reported similar findings in an RCT conducted in hazardous and harmful drinkers. Given the following: 1) power of 80%, 2) a two-sided 0.05 significance level, 3) a standard deviation for number of binge episodes in the past 28 days of 5.2, and 4) a 1:1

intervention allocation, a sample size of 327 subjects per group will be required to detect a 1.14 difference between AB-CASI and SC in the number of binge episodes in the past 28 days at 12 months. A total of 820 unhealthy drinkers will be enrolled and randomized to accommodate up to 20% dropout. To maximize the ability to explore modification by preferred language, we will enroll an equal number of preferred English (n=410) and Spanish (n=410) speaking Latinos.

6.2 Secondary and Exploratory Outcomes

Power calculations are provided for Aims 2 and 3 based on the sample size determined for Aim 1. Comparison of secondary outcomes between study groups will be evaluated at the two-sided 0.01 significance level to control inflated type I error from multiple significance testing. Our previous study[123,124] of at-risk individuals demonstrated a difference between subjects receiving a BNI compared to SC of 2.2 drinks per week at 12 months with a standard deviation of 7.5. In their review of brief behavioral counseling in primary care settings, Whitlock et al.[137] found that good quality brief intervention trials reduced consumption by 2.9 to 8.7 drinks per week. Similar conclusions were determined in a meta-analysis by Kaner et al.[138] The estimated sample size of 327 per group will provide 80% power to detect differences between intervention groups of 2.0 in the number of drinks per week. In general for continuous outcomes such as the SIP, the sample size above will allow for the detection of small effect sizes (d=0.27) with 80% power. For dichotomous secondary outcomes such as impaired driving, riding with an impaired driver, injuries or arrests, the study will have 80% power to detect absolute risk reductions of 13%, 10% and 7% for SC proportions with events of 50, 20 and 10% respectively.

For Aim 3, allowing for a 15% dropout at 30-days there will be close to 700 subjects that complete their 30-day Treatment Services Review follow-up. With a relatively low rate of expected 30-day treatment engagement in the SC group (conservatively, no greater than 20%), we'll have 80% power at the two-sided 0.01 significance level to detect an increase of 12% in the AB-CASI group.

7. INTERIM MONITORING

Interim monitoring will focus on safety, recruitment, adherence to protocol, baseline comparability of treatment groups, completeness of data retrieval, and uptake of the assigned intervention. A set of monitoring tables will be generated for this purpose (see DSMB Tables, Listings and Figures). No monitoring for efficacy or futility is being proposed.

7.1 Safety Monitoring

8. DATA COLLECTION AND DATA FREEZE

Data collected from date of study initiation (10/30/14) to last date of study follow-up (05/01/20) will be used. The trial database will be closed/frozen on (05/01/20) and be considered ready for final analysis.

9. ANALYSIS PLAN

This section describes the analysis of the primary, secondary and exploratory outcomes, including safety. The analytic plan will be modified as necessary up to final data lock and before study unblinding. Results will be reported to clinicaltrials.gov within one year of obtaining the primary outcome on the last patient. The trial has been registered on clinicaltrials.gov. Data analysis will be performed by the unblinded statisticians.

9.1 General Approach.

Nominal and ordinal categorical variables will be summarized using frequencies and percentages. Continuous variables will be summarized with the following descriptive statistics: N, mean, standard deviation, median, minimum, maximum, interquartile range, and range. No imputation of missing data will be performed in the primary and secondary analyses. Diagnostic tests and sensitivity analyses will be performed. Parametric distributional assumptions will be checked. If assumptions fail, other distributions will be considered prior to transformations and non-parametric methods.

9.2 Comparability of Baseline Characteristics.

Distributions of baseline demographic and clinical characteristics will be summarized by intervention group. Comparability for continuous variables will be examined graphically and by summary statistics (means, medians, quartiles, etc.). Categorical variables will be examined by calculating frequency distributions.

9.3 Analysis of Primary Outcomes

The primary objective of the analysis is to demonstrate that AB-CASI will reduce alcohol consumption more than SC in unhealthy drinkers at 12 months. The primary outcome (number of binge episodes in past 28-days) will be assessed using the 28-day timeline follow-back method at baseline (prior to initiation of intervention), 1-, 6- and 12-months after the intervention initiation. Likelihood-based ignorable analysis using a mixed model will be used to compare alcohol consumption between groups.[139,140] The primary advantage of the mixed model when compared to commonly used methods such as complete case analysis and single imputation (e.g. last observation carried forward) is its flexibility in handling missing data. This analysis will assume that missing data occurs at random (i.e. MAR, not informative). The inclusion of baseline 6- and 12-months outcome data in the model will assist in meeting this assumption. Furthermore, we will evaluate patterns of missing data as well as determine baseline characteristics that are predictive of dropout. If identified, these characteristics will be included in the model to meet the MAR assumption. A generalized linear mixed model (GLMM) with a Negative Binomial distribution will be used to estimate differences in the number of binge drinking episodes in the past 28 days. More specifically the mixed model will include fixed effects for intervention (AB-CASI vs. SC), time (1, 6, 12 months), and the interaction of intervention with time. Additional fixed effects will be included for baseline covariates (baseline number of drinks per week, baseline number of binge episodes, gender, English vs. Spanish preferred language and dependence status). Modification of the intervention effect by preferred language will be evaluated at the 0.10 significance level by including two and three-way interactions of language with intervention and time. If not significant, these interactions will be excluded and intervention effects pooled across preferred language strata. Similar procedures will be used to assess modification by dependence status. Linear contrasts (at the 0.05 two-sided significance level) will be used to estimate intervention group differences and 95% confidence intervals at the 1-, 6- and 12-month time points. Using a linear mixed effects model,

a similar analysis will be performed for the secondary outcome mean number of drinks per week over the last 28-days.

9.3.1 Heterogeneity of treatment effects (HTE)

HTE on the primary outcome will be assessed for subgroups based on factors assessed at baseline (Latino ancestry, age, birthplace, gender, preferred language, reason for ED visit and smoking status). These subgroup analyses will be conducted within the Generalized Linear Mixed Model framework in an evaluation similar to that proposed for investigating modification by the stratification factors of dependence status and preferred language as described above. Significant interactions will be followed by the estimation and summarization of intervention effects within subgroups at both 1-, 6- and 12-month time points.

9.4 Analysis of Secondary Outcomes

The primary objective of Aim 2 is to demonstrate that number of drinks per week and negative behaviors and consequences (episodes of impaired driving, riding with an impaired driver, injuries, arrests, tardiness, days absent from work/school and SIP) during the 12-month follow-up will be improved in subjects receiving AB-CASI compared to those receiving SC. Similar repeated measures mixed model analysis as that specified for the primary outcome will be implemented for each of the deleterious outcomes assessed using the Brief Event Data survey. Comparison of all secondary outcomes between study groups will be evaluated at the two-sided 0.01 significance level to control inflated type I error from multiple significance testing.

9.5 Analysis of Tertiary Outcomes

The analysis of tertiary outcomes will be considered exploratory and, thus, no control for multiplicity will be done. The objective of Aim 3 is to determine the effect of the AB-CASI compared to SC on 30-day treatment engagement. We hypothesize that AB-CASI will be superior to SC. Mantel-Haenszel chi-square analysis will be used to compare the likelihood of 30-day treatment engagement in AB-CASI to SC while adjusting for preferred language and dependence status. Significance will be judged at the two-sided 0.05 significance level. Heterogeneity of treatment effect will be evaluated by the Breslow-Day test. Participants dropping out or lost-to-follow-up will be considered to be not engaged in treatment for the primary analysis.

9.6 Missing Data

Several strategies will be imposed to accommodate the likelihood that missing data will occur during this study. Prevention is the most obvious and effective manner to control bias and loss of power from missing data.[141] This protocol will follow the intent to treat principle, requiring follow-up of all subjects randomized regardless of the actual treatment received.[142] Telephone visit reminders will be delivered to participants prior to protocol specified collection times. Alternative contact information will be identified on entry into the study to minimize loss-to follow-up. Timely data entry combined with weekly missing data reports will trigger protocols for tracking and obtaining missing data items or outcome assessments.

Despite these prevention efforts it is reasonable to assume missing data will occur. Our proposed primary and secondary analyses make use of all available data and are valid under the assumption that missing data will be missing at random (MAR) (Diggle, et al., 2002; Molenberghs et al., 2004). We will evaluate the plausibility of this assumption by determining the

extent of missing data and using logistic regression to identify factors associated with dropout. While we do not expect differential rates of dropout between groups or high loss to follow-up, if appropriate, we will conduct sensitivity analysis using a pattern-mixture approach implemented using multiple imputation under missing not at random (MNAR) assumptions to examine the robustness of conclusions of the primary analysis to missing data (National Research Council, 2010; Molenberghs et al., 2004).

REFERENCES

Callahan CM, Boustani MA, Unverzagt FW, Austrom MG, Damush TM, Perkins AJ, Fultz BA, Hui SL, Counsell SR, Hendrie HC. Effectiveness of collaborative care for older adults with Alzheimer disease in primary care: a randomized controlled trial. Journal of the American Medical Association. 295(18):2148-57,2006. PubMed PMID: 16684985.

Diggle PJ, Heagerty P, Liang K-Y, Zeger S. Analysis of Longitudinal Data. Oxford Science publications, Clarendon Press, Oxford, 2002 (2nd ed.)

Efird JT, Nielsen SS. Method to compute multiplicity corrected confidence intervals for odds ratios and other relative effect estimates. International Journal of Environmental Research and Public Health. 5(5) 394-398, 2008.

FDA Guidance for Industry. Oversight of Clinical Investigations - A Risk-Based Approach to Monitoring. August 2013.

Henderson R, Diggle P, Dobson A. Joint modeling of longitudinal measurements and event time data. Biostatistics.1:465–480, 2000.

Hochberg Y. A sharper Bonferroni procedure for multiple tests of significance. Biometrika. 75:800-802, 1988.

Lachin JM. Statistical considerations in the intent-to-treat principle. Controlled Clinical Trials. 21:167-89, 2000.

Mao HF, Kuo CA, Huang WN, Cummings JL, Hwang TJ. Values of the Minimal Clinically Important Difference for the Neuropsychiatric Inventory Questionnaire in Individuals with Dementia. Journal of the American Geriatric Society. 63(7):1448-52, 2015. doi:10.1111/jgs.13473. Epub 2015 Jun 5. PubMed PMID: 26046666.

Molenberghs G, Thijs H, Jansen I, et al. Analyzing incomplete longitudinal clinical trial data. Biostatistics. 5(3):445-64, 2004.

National Research Council. The Prevention and Treatment of Missing Data in Clinical Trials. Panel on Handling Missing Data in Clinical Trials. Committee on National Statistics, Division of Behavioral and Social Sciences and Education. Washington, DC: The National Academies Press, 2010.

Reuben DB. Final Progress report for 1C1CMS330982 “UCLA Alzheimer’s and Dementia Care: Comprehensive coordinated, patient-centered” submitted to CMS March 31, 2016.
