# Supplementary material for: Use of an Automated Bilingual Digital Health Tool to Reduce Unhealthy Alcohol Use Among Latino Emergency Department Patients: A Randomized Clinical Trial
Source: JAMA Netw Open. 2023 May 23;6(5):e2314848. doi: 10.1001/jamanetworkopen.2023.14848 (PMC10208138; doi:10.1001/jamanetworkopen.2023.14848)
Supplement: Supplement 2. — eFigure. Example Screenshots of AB-CASI: English and Spanish Versions [file jamanetwopen-e2314848-s002.pdf]

Supplementary Online Content

Vaca FE, Dziura J, Abujarad F, et al. Use of an automated bilingual digital health tool to reduce unhealthy alcohol use among Latino emergency department patients: a randomized clinical trial. *JAMA Netw Open*. 2023;6(5):e2314848. doi:10.1001/jamanetworkopen.2023.14848

eFigure. Example Screenshots of AB-CASI: English and Spanish Versions

This supplemental material has been provided by the authors to give readers additional information about their work.

© 2023 Vaca FE et al. *JAMA Network Open*.

eFigure. Example Screenshots of AB-CASI: English and Spanish Versions

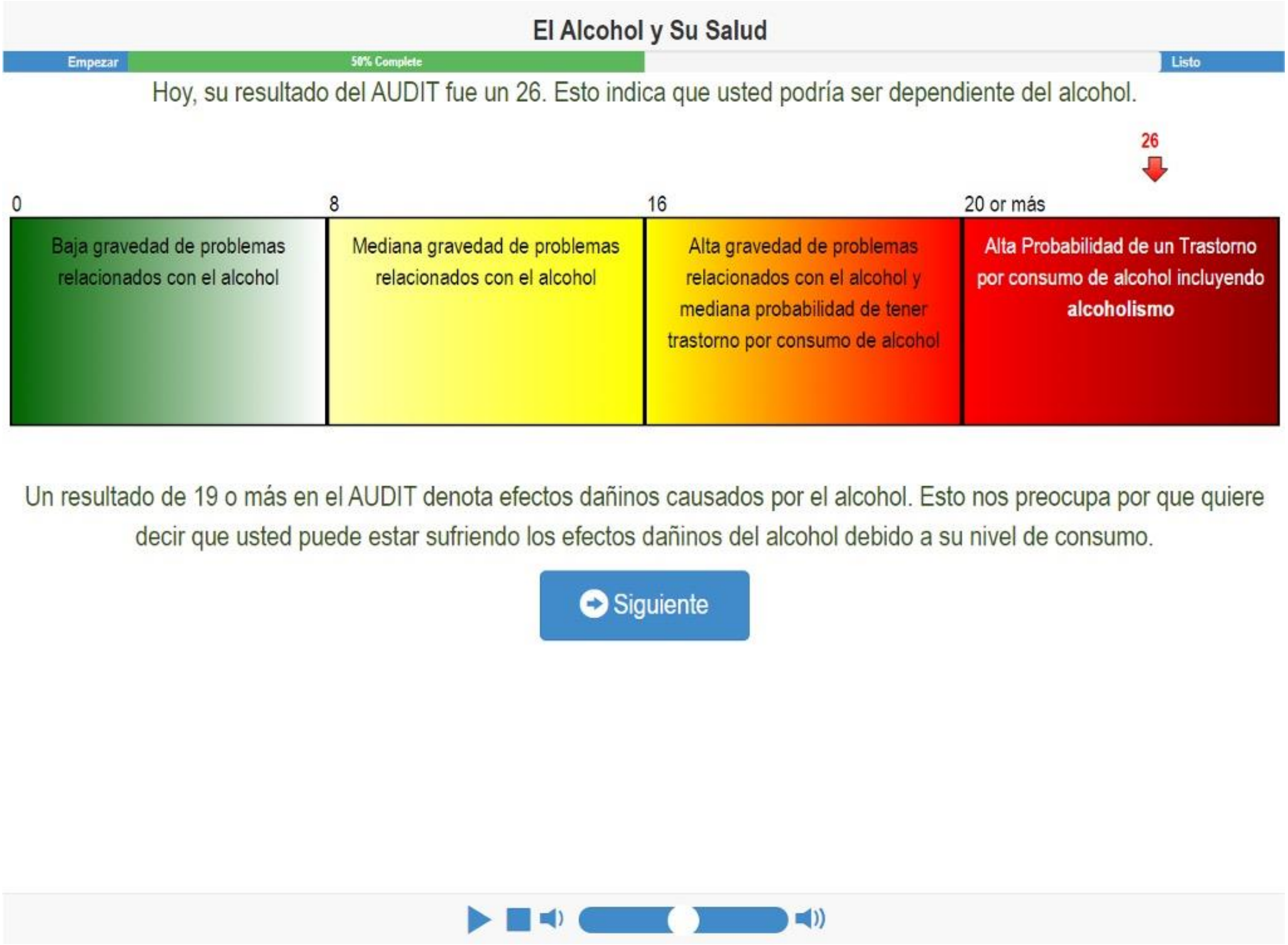

Panel 1. Spanish - AUDIT score presented with reference to severity levels of potential alcohol health effects

© 2023 Vaca FE et al. *JAMA Network Open*.

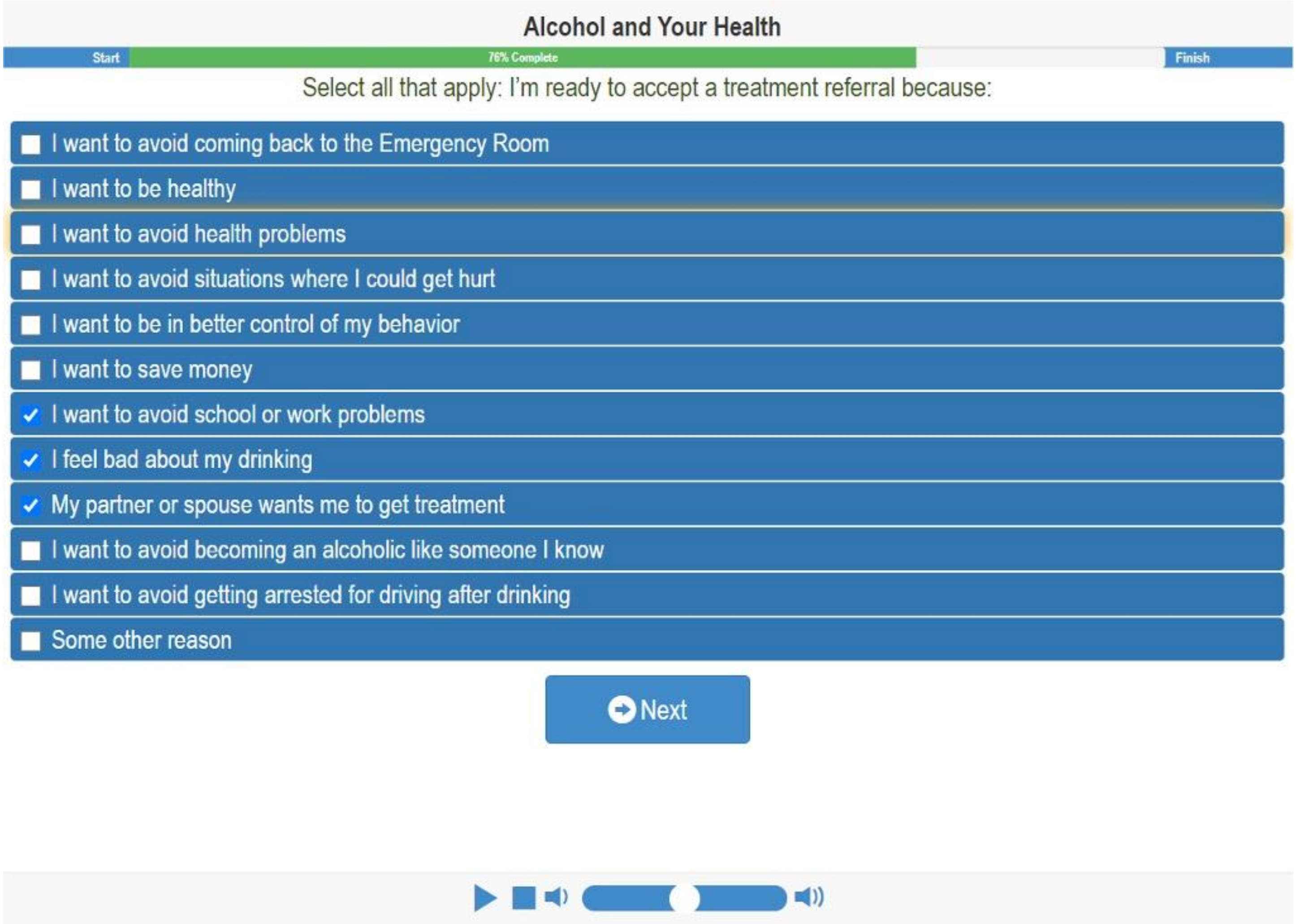

Panel 2. English - Listed reasons for a participant of higher alcohol use disorder severity to choose a reason(s) they are ready to accept a specialized treatment referral today

© 2023 Vaca FE et al. *JAMA Network Open*.

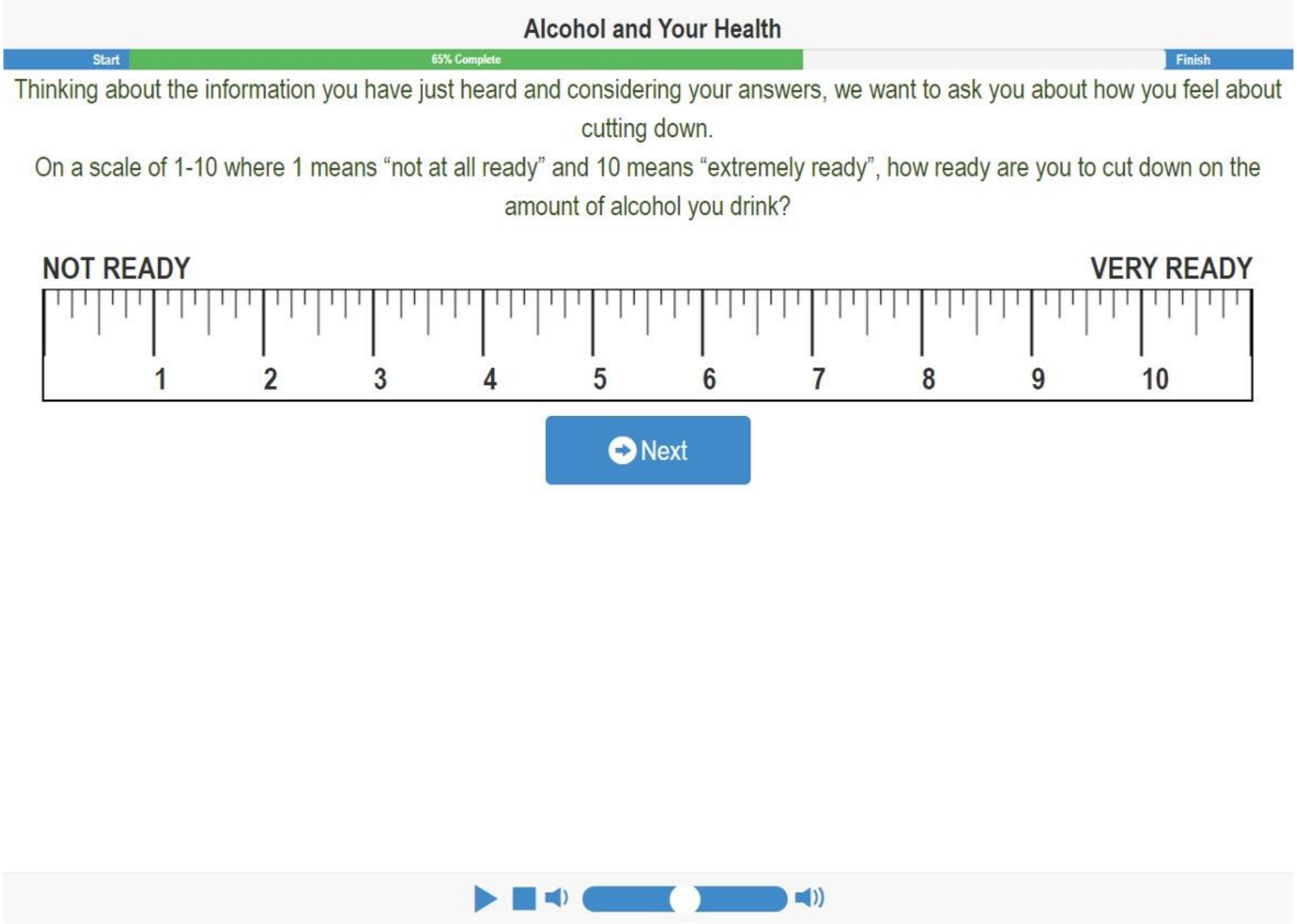

Panel 3. English - Readiness to change ruler

© 2023 Vaca FE et al. *JAMA Network Open*.

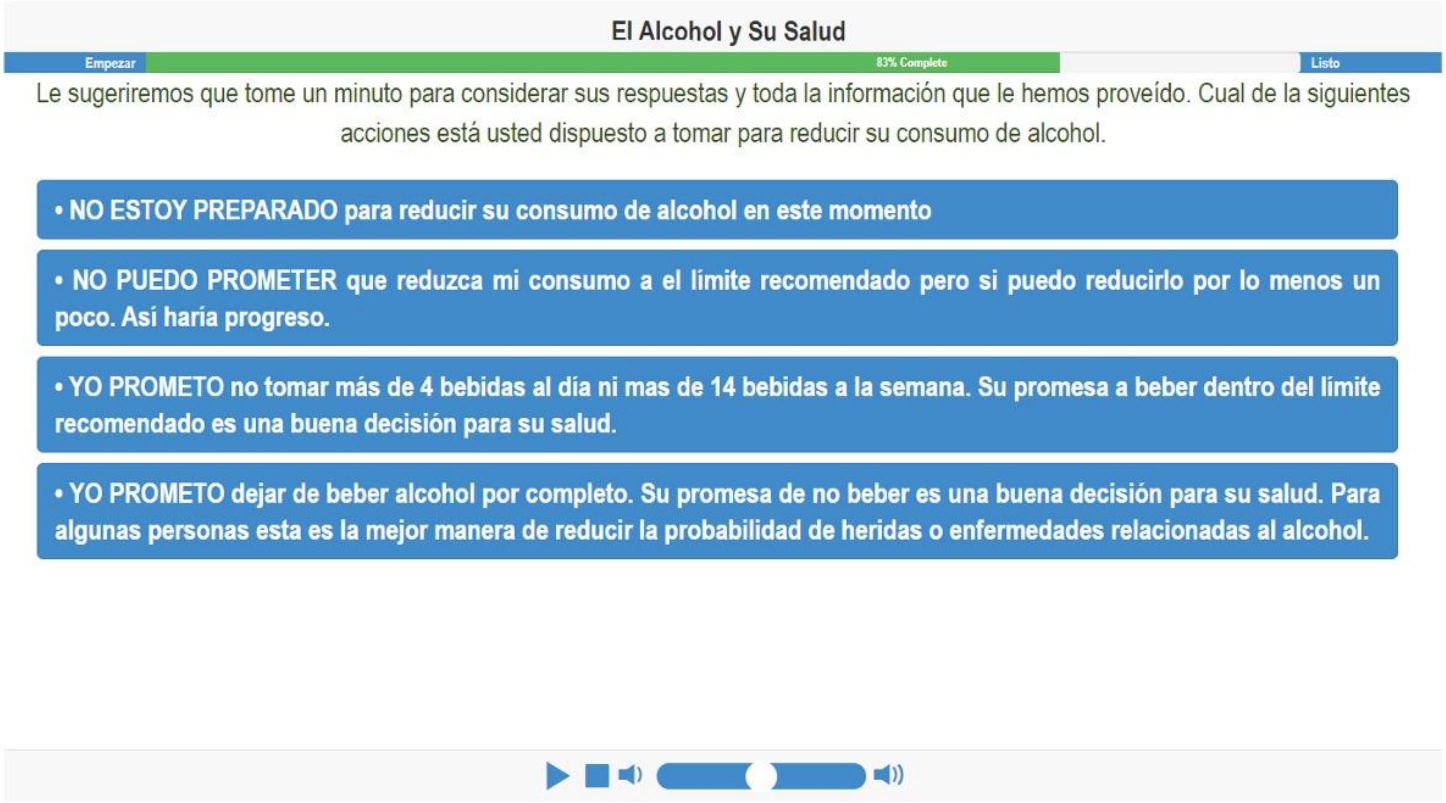

Panel 4. Spanish - Four actions for a participant of lower alcohol use disorder severity to choose from as their own next step as they consider their health and alcohol use

© 2023 Vaca FE et al. *JAMA Network Open*.
