# Supplementary material for: Use of an Automated Bilingual Digital Health Tool to Reduce Unhealthy Alcohol Use Among Latino Emergency Department Patients: A Randomized Clinical Trial
Source: JAMA Netw Open. 2023 May 23;6(5):e2314848. doi: 10.1001/jamanetworkopen.2023.14848 (PMC10208138; doi:10.1001/jamanetworkopen.2023.14848)
Supplement: Supplement 3. — Data Sharing Statement [file jamanetwopen-e2314848-s003.pdf]

Data Sharing Statement

Vaca. Use of an Automated Bilingual Digital Health Tool to Reduce Unhealthy Alcohol Use Among Latino Emergency Department Patients. *JAMA Netw Open*. Published May 23, 2023. doi:10.1001/jamanetworkopen.2023.14848

Data
Data available: Yes
Data types: Other (please specify)
Additional Information: Data used specifically in this manuscript could be made available to research scientists upon review of request and in accordance with privacy policies
How to access data: Initially contact corresponding author
When available: With publication

Supporting Documents
Document types: None

Additional Information
Who can access the data: Research scientists whose proposed use of the data has been approved in accordance with all policies
Types of analyses: Secondary analysis of data used specifically in this manuscript
Mechanisms of data availability: After approval of an IRB proposal and with a signed and executed data use agreement
